# A Global Perspective on Cardiovascular Risk Factors by Educational Level in CHD Patients: SURF CHD II

**DOI:** 10.5334/gh.1340

**Published:** 2024-07-16

**Authors:** Anna Marzà-Florensa, Ilonca Vaartjes, Ian Graham, Kerstin Klipstein-Grobusch, Diederick E. Grobbee, Marina Joseph, Yanina Castillo Costa, Nicolás Esteybar Enrique, Rahima Gabulova, Mahluga Isaveva, Farid Alivev, Uzeyir Rahimov, Galib Imanov, Firdovsi Ibrahimov, Zarbaliyeva Naila, Rashad Abasov, Paul Dendale, Anre Jassen, Johan De Sutter, Sofie Cuypers, Dalton Precoma, Luiz Ritt, Mario Claudio Soares Sturzeneker, Conrado Roberto Hoffmann Filho, Maria Teresa Lira, Michal Varablik, Eva Tumova, Jaromir Ozana, Ann Bovin, Margus Viigimaa, Konstantinos Tsioufis, Ageliki Laina, Zacharoulis Achilles, Fotios Toulgaridis, Elias Sanidas, Zeljko Reiner, Marijana Gulin, Antonijo Bejúić, Darko Duplančić, Jozica Šikić, Eszter Szabados, Badai Bhatara Tiksnadi, Bill McEvoy, Anne Reynolds, David Moore, Declan Spelman, Raman Puri, Rashmi Nanda, Nagaraj Desai, Prabhakar Dorairaj, S. S. Iyengar, Sumitra Lakshmana, Ajay Kumar Pandey, Akshyaya Pradhan, Kunal Mahajan, Pompilio Faggiano, P. Zarcone, Maurizio G. Abrignani, Makhanov Daniyar, Kairat Davletov, Kuatbek Toleubekov, Olga Visternichan, Alibek Mereke, Anar Dushpanova, Bekbolat Zholdin, Zhanat Timirbayev, Gulmira Derbissalina, Daulet K. Aldyngurov, Ayan Myssayev, Alma Nurtazina, Zhanna Romanova, Sundetgali Kalmakhanov, Iveta Mintale, Omar Msalam, Emhemed Ehemmali, Alami Mohamed, Najat Mouine, Tazi Mezalek Amale, Aida Soufiani, Zineb Agoumy, Houda Bachri, Imad Massri, Irena Mitevska, Saskia Haitjema, Mark de Groot, Ana Abreu, Svetlana Mosterou, Dan Gaita, Nana Pogosova, Marat V. Ezhov, Abdulhalim Kinsara, Ivana Burazor, Vojislav Giga, Hector Bueno, Regina Dalmau, Ana García-Barrios, Jose Antonio Alarcon Duque, Joana Reparaz Mendinueta, Meral Kayikcioglu, Lale Tokgozoglu, Emre Aslanger, Ayca Turer Cabbar, Samuel Kim, Caleb Self, Dolores Reynolds, Sabrina Rose, Pretti Singh

**Affiliations:** 1 Julius Global Health, Julius Center for Health Sciences and Primary Care, University Medical Center Utrecht, Utrecht University, Utrecht, The Netherlands; 2 Trinity College, Dublin, Ireland; 3 Division of Epidemiology and Biostatistics, School of Public Health, Faculty of Health Sciences, University of the Witwatersrand, Johannesburg, South Africa; 4 Sociedad Argentina de Cardiología, Buenos Aires, Argentina; 5 Counsel of Cardioecology and Healthy Habits Sociedad Argentina de Cardiología, Mar del Plata, Argentina; 6 Azerbaijan Medical University, Educational Therapeutic Clinic, Baku, Azerbaijan; 7 Scientific Research Institute for Cardiology, Baku, Azerbaijan; 8 Baku Health Centre, Baku, Azerbaijan; 9 Baku Medical Plaza, Baku, Azerbaijan; 10 Azerbaijan Medical University, Educational Surgical Clinic, Baku, Azerbaijan; 11 Central Clinic Hospital, Baku, Azerbaijan; 12 Ganja City United Hospital, Ganja, Azerbaijan; 13 ER Medical, Khachmaz, Azerbaijan; 14 Jessa Hospital-Hartcentrum, Hasselt, Belgium; 15 AZ Maria Middelares Cardiologie, Gent, Belgium; 16 Hartcentrum OLV ziekenhuis, Aalst, Belgium; 17 Sociedade Hospitalar Angelina Caron, Campina Grande do Sul, Brazil; 18 Escola Bahiana de Medicina e Saúde Pública, Salvador, Brazil; 19 Wallace Thadeu de Mello e Silva Regional University Hospital, Brazil; 20 Street Blumenau, 294 Centro, Joinville SC, Brazil; 21 Hospital Clínico Fuerza Aérea de Chile, Santiago, Chile; 22 Centre of Preventive Cardiology, 3rd Department of Internal Medicine, 1st Faculty of Medicine and General Teaching Hospital, Prague, Czech Republic; 23 Department of Exercise Medicine and Cardiovascular Rehabilitation, University Hospital Olomouc, Czech Republic; 24 Danish Society of Cardiology, Copenhagen, Denmark; 25 North Estonia Medical Centre, Tallinn University of Technology, Tallin, Estonia; 26 Cardiology Department, Hippokration General Hospital, Athens, Greece; 27 General Hospital of Athens “Evangelismos”, Athens, Greece; 28 LAIKO General Hospital, Athens, Greece; 29 Polish Mother’s Memorial Hospital Research Institute, Lodz, Poland & University Hospital Center, Zagreb, Croatia; 30 County Hospital Sibenik, Croatia; 31 University Hospital, Split, Croatia; 32 University Hospital Sveti Duh, Croatia; 33 1st Department of Medicine, Division of Preventive Cardiology and Rehabilitation, University of Pécs, Medical School, Pécs, Hungary; 34 Hasan Sadikin General Hospital, Bandung, West Java, Indonesia; 35 University Hospital Galway, Galway, Ireland; 36 Tallaght University, Hospital Cardiology clinic, Dublin, Ireland; 37 South Tipperary General Hospital, Tipperary, Ireland; 38 Cardiac Care Centre, New Delhi, India; 39 Namana Medical Centre, Bengaluru, India; 40 Ashwin Clinic, Annanagar, India; 41 Manipal Hospital, Bangalore, India; 42 Galaxy Hospital, Mahmoorganj, India; 43 King George’s Medical University, Lucknow, India; 44 Himachal Heart Institute, Chhanwari, India; 45 Ospedali Riuniti, Brescia, Italy; 46 Policlinico P. Giccone, Palermo, Italy; 47 S Antonio Abate Hospital of Trapani, O.U. of Cardiology, Trapani, Italy; 48 Central Clinical Hospital, Almaty, Kazakhstan; 49 Asfendiyarov Kazakh National Medical University, Almaty, Republic of Kazakhstan; 50 Cardiorehabilitation Center “Tulpar”, Karaganda Medical University, Karaganda, Kazakhstan; 51 City Policlinic № 32, Al Farabi Kazakh National University, Almaty, Kazakhstan; 52 Scuola Superiore Sant’Anna, Pisa, Italy; 53 Medical Center of West Kazakhstan Marat Ospanov Medical University, Zhanakonys, Kazakhstan; 54 Astana Medical University, University hospital, Astana, Kazakhstan; 55 Department of Science and Human resource, Ministry of Healthcare, Kazakhstan; 56 Cardiological Center of Pavlodar region, Department of Science and Human Resource, Ministry of Healthcare, Kazakhstan; 57 Cardiological Center of Pavlodar region, Semey Medical University, Semey, Kazakhstan; 58 City polyclinic № 5, Al Farabi Kazakh National University, Almaty, Kazakhstan; 59 Latvian Center of Cardiology, Riga, Latvia; 60 Libyan Cardiac Society, Tripoli, Libya; 61 Misrata Heart & Cardiovascular Center, Misrata, Libya; 62 Cabinet Cardiologie ALAMI, Casablanca, Morocco; 63 Cardiology Centre, Mohammed V Military Hospital, Rabat, Morocco; 64 Hopital Universitaire Cheikh Zaid, Rabat, Morocco; 65 Ligue Nationale de cardiologie, Rabat, Morocco; 66 University Cardiology Clinic, Skopje, North Macedonia; 67 Utrecht Patient Oriented Database (UPOD), Central Diagnostic Laboratory, Division of Laboratory, Pharmacy, and Biogenetics, University Medical Center Utrecht, Utrecht University, Utrecht, The Netherlands; 68 Hospital Universitário Santa Maria, IMPSP, ISAMB, Faculty of Medicine of University of Lisbon, Lisbon, Portugal; 69 Institute for Cardiovascular Diseases, Timisoara, Romania; 70 National Medical Research Center of Cardiology, Preventive Cardiology Laboratory, Moscow, Russia; 71 Federal State Budget Institution, National Cardiology Research Centre of Ministry of Healthcare of Russian Federation, Moscow, Russia; 72 Ministry of National Guard Health Affairs, King Saud bin Abdulaziz University for Health Sciences, COM-WR, King Abdullah International Research Center, Jeddah, Saudi Arabia; 73 Cardiology, Institute for Rehabilitation, Serbia; 74 University of Belgrade – Faculty of Medicine, Institute for Cardiovascular Diseases ‘Dedinje’, Belgrade, Serbia; 75 Cardiology Clinic, Clinical Center of Serbia, Belgrade, Serbia; 76 Hospital 12 de Octubre, Madrid, and Centro Nacional de Investigaciones Cardiovasculares, Madrid, Spain; 77 University Hospital la Paz, Madrid, Spain; 78 Hospital General Universitario Dr. Balmis, Alicante, Spain; 79 Hospital Universitario Donostia/OSI Donostialdea, Donostia, Spain; 80 Ege University Medical School Cardiology Department, Ege University Tip Fak Kardivoloii AD Bornova Izmir, Turkey; 81 Hacettepe University, Department of Cardiology, Ankara, Turkey; 82 Basaksehir Pine and Sakura City Hospital, Istanbul, Turkey; 83 Yeditepe University Hospital, Istanbul, Turkey; 84 Weil Cornell Medicine, New York, United States

**Keywords:** Secondary prevention, coronary heart disease, health inequities, education, cardiovascular risk factors, global health

## Abstract

**Background:**

Clinical guidelines recommend lifestyle modifications and medication use to control cardiovascular risk factors in coronary heart disease (CHD) patients. However, risk factor control remains challenging especially in patients with lower educational level.

**Objective:**

To assess inequalities by educational level in the secondary prevention of CHD in the Survey of Risk Factors in Coronary Heart Disease (SURF CHD II).

**Methods:**

SURF CHD II is a cross-sectional clinical audit on secondary prevention of CHD, conducted during routine clinical visits in 29 countries. The easy-to-perform design of the survey facilitates its implementation in settings with limited resources. We reported risk factor recording, attainment of guideline-defined risk factor targets, and treatment in CHD patients. Differences by educational level in target attainment and treatment were assessed with logistic regression stratified for high- (HIC), upper middle- (UMIC), and lower middle-income (LMIC) countries.

**Results:**

SURF CHD II included 13,884 patients from 2019 to 2022, of which 25.0% were female and 18.6% had achieved only primary school level. Risk factor recording ranged from 22.2% for waist circumference to 95.6% for smoking status, and target attainment from 15.9% for waist circumference to 78.7% for smoking. Most patients used cardioprotective medications and 50.5% attended cardiac rehabilitation.

Patients with secondary or tertiary education were more likely to meet targets for smoking, LDL cholesterol and physical activity in HICs and LMICs; for physical activity and triglycerides in UMICs; but less likely to meet targets for blood pressure in HICs and LDL <1.4mmol/L in UMICs. Higher education was positively associated with medication use and cardiac rehabilitation participation.

**Conclusion:**

CHD patients generally have poor attainment of risk factor targets, but patients with a higher educational level are generally more likely to participate in cardiac rehabilitation, use medication, and meet targets.

**Main messages:**

1. Target attainment and participation in cardiac rehabilitation are poor in CHD patients globally.
2. Patients with higher education are more likely to meet risk factor targets, showing health inequities in secondary prevention of CHD.
3. The association between education and risk factor target attainment and treatment varies with country income level.

## Introduction

Coronary heart disease (CHD) is the leading cause of death and a major cause of disability worldwide [1]. The Global Burden of Disease Study estimates that there were 9.14 million deaths and 197 million prevalent cases of CHD in 2019 occurring predominantly in low- and middle-income countries (LMIC) [2]. Most of the burden of CHD is attributable to risk factors, many of which are potentially modifiable or controllable through lifestyle changes and medication use [2,3,4].

People with established CHD are at very high cardiovascular risk [5]. Therefore, risk factor control through lifestyle modification and medical therapy is fundamental to reduce risk of cardiovascular events and mortality. Clinical guidelines define targets for risk factor control in CHD patients, including smoking cessation, physical activity, weight and body composition, blood pressure, lipids, and glucose levels (Table 1) [5,6].

**Table 1:** Definition of risk factor targets.


RISK FACTOR	TARGET

Smoking	No smoking or cessation

Physical activity	Moderately vigorous physical activity ≥30 minutes 3–5 times/week

BMI	<25 kg/m^2^

Waist circumference	<94 cm in men (<90 cm in South-East Asian men) and <80 cm in women

Blood pressure	<140/90 mmHg (<140/85 mmHg in diabetics)

LDL	<1.8 mmol/L. Stricter target: <1.4 mmol/L

non-HDL cholesterol	<2.2 mmol/L

Triglycerides	<1.7 mmol/L

Hba1c (in diabetics)	<7%


There are several challenges in secondary prevention of CHD. Risk factor data is often not recorded in full, and thus relevant information relevant for risk factor management may be unavailable in daily practice. In terms of control, large proportions of participants do not meet risk factor targets. The most recent EUROASPIRE survey [7] reported poor levels of target attainment, despite high rates of medication use. Research on the drivers of risk factor control in patients with CHD can provide a deeper understanding of the challenges to adequate secondary prevention.

Socioeconomic circumstances, such as educational level, have shown to have an impact on risk factor control and medication use in the context of secondary prevention. Lower educational level has been linked to higher prevalence of risk factors, lower treatment levels, and higher risk of future cardiovascular events [8,9]. Such inequalities in secondary prevention have shown to be context-dependent and may vary in different regions [10].

The Survey in Risk Factors in Coronary Heart Disease II (SURF CHD II) Study is an easy-to perform clinical audit designed to evaluate compliance with clinical guidelines in secondary prevention in daily practice. In this article, SURF CHD II data are used to assess secondary prevention of CHD and to investigate potential inequalities in risk factor management.

Specifically, we report the level of risk factor recording, guideline-defined target attainment, and treatment in CHD outpatients and investigate differences by educational level. Our results can bring attention to potential health inequalities aimed at supporting the development of effective prevention strategies.

## Methods

### Study design and data collection

SURF CHD II consists of brief cross-sectional survey that collects data on demographics, risk factor history, risk factor measurements, medications, and participation in cardiac rehabilitation. The survey is performed as part of a clinical audit in consecutive patients with CHD attending routine outpatient visits. Centers that registered ≥100 participants were included in the present analysis.

The survey was completed from 2019 to 2022 in 105 centers located in 29 countries, including high-income countries (HICs) (Belgium, Chile, Croatia, Czech Republic, Denmark, Estonia, Greece, Hungary, Ireland, Italy, Latvia, the Netherlands, Portugal, Romania, Saudi Arabia, Spain, and the United States of America), upper middle-income countries (UMICs) (Argentina, Azerbaijan, Brazil, Kazakhstan, Libya, North Macedonia, Russia, Serbia, and Turkey), and lower middle-income countries (LMICs) (India, Indonesia, and Morocco) [11].

Patient eligibility criteria was being 18 years or older and having a previous diagnosis of CHD, including stable angina pectoris (SAP), acute coronary syndrome (ACS), percutaneous coronary intervention (PCI) (elective or acute) and/or coronary artery bypass graft (CABG) (elective or acute). SAP is defined as clinical angina with objective confirmation from ECG, ischemia on perfusion imaging, coronary angiogram showing a narrowing of 70% in at least one coronary artery.

### Data sources

Data was obtained from medical records and patient interview by a physician or nurse. Participating centers in Denmark, Italy, the Netherlands, and the United States, extracted data from existing health registries of patients who were eligible for the study [12,13]. Data was collected by use of the software RedCap [14].

### Ethical considerations

Ethical approval for this study was waived by the Medical Ethics Committee of the University Medical Center Utrecht (protocol number 17/534). Ethical approval was obtained or waived in individual participating centers prior to participation.

### Data collection and variable definition

We registered center-level information, including the type of center (public or private) and location (urban or rural area). Routine patient data were collected on age, sex, ethnicity, educational level, and CHD diagnostic category. Ethnicity was classified as Arab, Asian, Black, Mixed, White, or other. Educational level was defined as the highest level achieved by participants and grouped as primary vs. secondary or tertiary education (including bachelor’s degree or higher technical certificate). CHD Diagnostic category included stable SAP, ACS, PCI, and CABG. Information on risk factor history included admission in the hospital for a CHD-related reason in the past year, smoking history, known history of hypertension, dyslipidemia, or diabetes. The survey included questions on whether patients had participated in a cardiac rehabilitation program, and if they were using the following medications: antiplatelet drugs, beta-blockers, ACE-inhibitors, ARBs, Ca antagonists, other antihypertensives, diuretics, statins, Pcsk9-inhibitors, other lipid-lowering medications, insulin, other hypoglycemics, or nitrates. Information on height and risk factor measurements performed up to three months prior to the visit, including systolic blood pressure, diastolic blood pressure, heart rate, height, weight, and waist circumference, were collected. The following fasting blood values from up to a year before the visit were registered: total cholesterol, LDL, HDL, triglycerides, glucose, and Hba1c in diabetics.

### Outcomes

Recording was defined as information available from interview, medical records, or laboratory results during the visit following the routine procedures. Given that one of the goals of the study was to assess risk factor recording in daily practice, health professionals were asked not to perform additional measurements outside routine care for the purpose of the survey. If a value was missing, marked “unknown” or not available in the original data source, we considered it not to be recorded.

Risk factor targets were defined according to European Society of Cardiology (ESC) clinical guidelines [5,6] (Table 1).

Treatment outcomes were defined as self-reported use of antiplatelet medication, antihypertensives (beta-blockers, ACE-inhibitors, ARBs, Ca antagonists, other antihypertensives), lipid-lowering medication (statins, Pcsk9-inhibitors, other lipid-lowering medications), insulin, oral hypoglycemics, and participation to cardiac rehabilitation.

### Data analysis

Categorical variables were presented as percentage of participants, and numerical variables as mean (standard deviation). We calculated the proportion of participants with recorded risk factor information, meeting risk factor targets, using medication, and participating in cardiac rehabilitation treatment by educational level.

We tested potential differences in risk factor recording, target attainment and medication use in patients with primary education compared with those with secondary or tertiary education using logistic regression adjusted by age and sex. Results are presented as odds ratios and 95% confidence intervals.

All analyses were stratified by region and performed with R Studio (version 4.0) [15]. Statistical significance was considered at a two-sided p < 0.05.

## Results

### Study population

A total of 13,884 CHD patients were included in the survey, of which about half were registered in a HIC center (N = 7462). 25.0% were female, and mean age was 64.8 (sd 11.2) years. Most participants were considered ethnically white (60.9%) and Asian (31.8%). With respect to educational level, 47.0% had completed tertiary education, 34.5% secondary school, and 18.6% primary school (Table 2). Mean systolic blood pressure was 132 (sd 19.0) mmHg, mean diastolic blood pressure was 77.9 (sd 11.1) mmHg, mean BMI was 28.0 (sd 4.9) kg/m^2^, and mean LDL cholesterol 2.24 (sd 1.1) mmol/L Supplementary Tables 1 and 2 show patient characteristics, risk factor history and measurements, fasting blood lipid and glucose levels, recording of risk factors, target attainment and treatment by HICs, UMICs, and LMICs–categorizing them by educational level. For 34.1% of the patients, data from pre-existing registries was used to complete the survey.

**Table 2:** Characteristics of the study population by country income group.


	(LMIC N = 2645)	UMIC (N = 3777)	HIC (N = 7462)	(Total N = 13884)

Number of countries	3	9	17	29

Number of centres	11	29	65	105

Type of centre attended				

Private	1526 (57.7)	940 (24.9)	313 (4.55)	2779 (20.9)

Public	1119 (42.3)	2837 (75.1)	6561 (95.4)	10517 (79.1)

Demographics				

Mean age (SD)	62.1 (39.9)	63.0 (10.5)	66.7 (11.0)	64.8 (20.0)

Sex				

Female	502 (19.0)	1140 (30.2)	1823 (24.4)	3465 (25.0)

Ethnic Group				

Arab	346 (13.1)	102 (2.7)	123 (4.3)	571 (6.1)

Asian	2284 (86.4)	628 (16.6)	26 (0.9)	2938 (31.8)

Black	6 (0.2)	18 (0.5)	14 (0.5)	38 (0.4)

Mixed	5 (0.2)	41 (1.1)	9 (0.3)	55 (0.6)

Other	0 (0.0)	3 (0.1)	8 (0.3)	11 (0.1)

White	4 (0.2)	2981 (79.0)	2634 (93.6)	5619 (60.9)

Educational level				

Primary school	630 (24.8)	506 (16.8)	921 (16.7)	2057 (18.6)

Secondary school	964 (37.9)	1403 (46.6)	1451 (26.3)	3818 (34.5)

Tertiary/University	950 (37.3)	1100 (36.6)	3150 (57.0)	5200 (47.0)

Cardiovascular history				

Index event				

CABG	423 (16.0)	690 (18.3)	797 (15.7)	1910 (16.6)

PCI	1253 (47.4)	2197 (58.2)	2875 (56.6)	6325 (55.0)

Acute coronary syndrome	1034 (39.1)	1462 (38.7)	4128 (55.3)	6624 (47.7)

Stable angina pectoris	855 (32.3)	1539 (40.7)	2146 (28.8)	4540 (32.7)

Family history premature CVD	288 (10.9)	1184 (31.4)	819 (29.0)	2291 (24.8)

Risk factor history				

Hypertension	1359 (51.4)	3109 (82.3)	3162 (65.7)	7630 (67.9)

Dyslipidemia	1234 (46.7)	1974 (52.3)	3163 (67.5)	6371 (57.4)

Diabetes	1129 (29.2)	1136 (42.9)	1294 (34.3)	3559 (34.6)

Smoking				

Current	423 (16.3)	861 (23.7)	1541 (21.9)	2825 (21.3)

Former	442 (17.0)	1112 (30.6)	3129 (44.4)	4683 (35.3)

Never	1736 (66.7)	1661 (45.7)	2373 (33.7)	5770 (43.5)

Physical activity < 30 minutes 3–5 times/week	979 (38.0)	1690 (52.9)	1640 (43.6)	4309 (45.2)

Moderate	1259 (48.8)	1037 (32.5)	1451 (38.5)	3747 (39.3)

Physical activity > 30 minutes 3–5 times/week	340 (13.2)	465 (14.6)	673 (17.9)	1478 (15.5)

Risk factor levels (mean (SD))				

Systolic BP (mmHg) mean (SD)	128 (20.2)	134 (20.3)	132 (17.7)	132 (19.0)

Diastolic BP (mmHg) mean (SD)	75.8 (11.3)	80.7 (11.4)	77.2 (10.6)	77.9 (11.1)

Heart rate (bpm) mean (SD)	77.8 (13.6)	73.7 (12.9)	68.9 (12.2)	73.1 (13.3)

BMI (kg/m^2^) mean (SD)	26.5 (4.5)	28.7 (4.9)	28.2 (5.0)	28.0 (4.9)

Waist circumference (cm) mean (SD)	90.5 (15.4)	99.2 (12.7)	103 (13.1)	100 (13.7)

Total cholesterol (mmol/l) mean (SD)	3.86 (1.2)	4.70 (1.4)	3.98 (1.4)	4.19 (1.4)

LDL cholesterol (mmol/l) mean (SD)	2.15 (1.1)	2.79 (1.2)	2.06 (1.0)	2.24 (1.1)

HDL cholesterol (mmol/l) mean (SD)	1.11 (0.4)	1.19 (0.4)	1.22 (0.4)	1.18 (0.4)

Tryglicerides (mmol/l) mean (SD)	3.74 (1.9)	2.44 (2.0)	2.76 (1.9)	2.92 (2.0)

Fasting glucose (mmol/l) mean (SD)	7.63 (3.5)	6.78 (2.8)	6.44 (2.2)	6.88 (2.9)

HbA1C (%)	7.97 (1.7)	7.74 (1.7)	8.94 (11.1)	8.23 (6.5)


Results are indicated in number of participants (%) unless indicated. HICs: high-income counties, UMICs: upper-middle-income countries, LMICs: lower-middle income countries, CABG: Coronary Artery Bypass Graft, PCI: Percutaneous Coronary Intervention, CVD: cardiovascular disease.

### Risk factor recording

**Figure 1:**
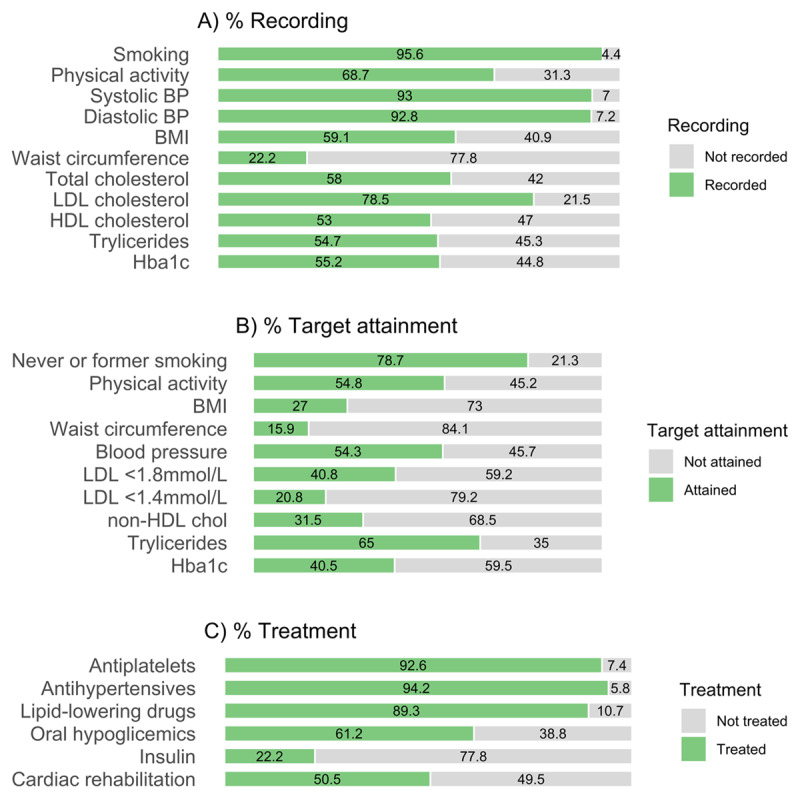
Percentage of participants **(A)** meeting risk factor targets, **(B)** with information recorded on risk factors, and **(C)** reporting to use medication and participate in cardiac rehabilitation. Risk factor targets are defined as: no smoking or smoking cessation, Moderately vigorous physical activity at least 30 minutes 3–5 times/week, BMI <25 kg/m^2^, waist circumference <94 cm in men (<90 cm in South-East Asian men) and <80 cm in women, blood pressure <140/90 mmHg (<140/85 mmHg in diabetics), LDL <1.8 mmol/L, LDL <1.4 mmol/L, non-HDL cholesterol <2.2 mmol/L, triglycerides <1.7 mmol/L, and Hba1c (in diabetic patients) <7%. Results on Hba1c recording, target attainment, oral hypoglicemics, and insulin are calculated among diabetic patients.

Risk factor recording was highest for smoking (95.6%) and blood pressure (92.8%), and lowest for waist circumference (22.2%). Other risk factors were recorded with variable frequency between 53.0% (HDL cholesterol) to 78.5% (LDL cholesterol) [Figure 1].

### Target attainment

More than three quarters (78.7%) of the study population met the target for smoking, as these either had never smoked or had quit smoking, and half (54.8%) was moderately or highly physically active. The risk factor measurement targets were met by 27.0% of the patients for BMI, 15.9% for waist circumference, and 54.3% for blood pressure. Regarding laboratory values, 40.8% had LDL levels <1.8 mmol/L, 20.8% LDL <1.4 mmol/L, 31.5% non-HDL-cholesterol <2.2 mmol/L, 65.0% triglycerides <1.7 mmol/L, and 40.5% of diabetic patients had Hba1c <7% [Figure 1].

In general, patients with a higher educational level were more likely to meet risk factor targets, though the associations between educational level and target attainment varied by risk factor and country income level. Patients with secondary or tertiary education were more likely to meet targets for smoking, physical activity, and LDL <1.8 mmol/L in HICs and LMICs, but they were less likely to meet risk the blood pressure target (in HICs). In UMICs, patients with secondary or tertiary education were more likely to meet physical activity and triglycerides targets; yet, they were less likely to have LDL <1.4 mmol/L (Table 3).

**Table 3:** Results multivariable analysis showing odds ratios for achieving risk factor targets, being on medication or participating in cardiac rehabilitation, and secondary or tertiary educational level compared to primary education.


	HIC	UMIC	LMIC

Target attainment^a^			

Never or former smoking	1.71 (1.43–2.04)	1.14 (0.88–1.47)	1.99 (1.53–2.59)

Physical activity	1.94 (1.59–2.35)	2.48 (1.96–3.14)	1.75 (1.41–2.13)

BMI	1.06 (0.84–1.34)	1.10 (0.81–1.51)	0.89 (0.71–1.11)

Waist circumference	1.30 (0.82–2.12)	0.63 (0.37–1.10)	0.86 (0.53–1.40)

Blood pressure	0.64 (0.53–0.77)	1.02 (0.83–1.27)	0.92 (0.75–1.14)

non-HDL Cholesterol	1.01 (0.79–1.30)	0.92 (0.64–1.35)	1.19 (0.94–1.49)

LDL 1.8	1.23 (1.05–1.45)	0.74 (0.55–1.01)	1.30 (1.04–1.63)

LDL 1.4	0.94 (0.78–1.13)	0.65 (0.44–1.00)^c^	1.21 (0.93–1.58)

Triglycerides	0.85 (0.66–1.09)	1.32 (1.01–1.72)	1.14 (0.91–1.44)

Hba1c^b^	0.82 (0.45–1.36)	0.89 (0.53–1.50)	1.20 (0.80–1.82)

Treatment			

Antiplatelets	1.66 (1.22–2.23)	1.285 (0.97–1.69)	1.5 (0.94–2.35)

Antihypertensives	1.995 (1.32–2.96)	0.72 (0.48–1.05)	1.03 (0.64–1.63)

Lipid-lowering	1.424 (1.05–1.90)	0.62 (0.48–0.79	1.75 (1.20–2.55)

Oral hypoglicemics	1.43 (0.94–2.16)	1.08 (0.78–1.49)	1.26 (0.92–1.72)

Insulin^b^	1.10 (0.70–1.73)	0.43 (0.30–0.61)	0.88 (0.59–1.34)

Cardiac rehabilitation^b^	2.47 (2.10–2.91)	4.89 (3.44–7.18)	1.24 (0.99–1.56)


Results are expressed in odds ratios (95% confidence intervals) of achieving risk factor targets and being on medication or participating in cardiac rehabilitation, if having secondary or tertiary education compared to primary education, adjusted by age, sex, and type of center (public or private). ^a^ Risk factor targets are defined as: no smoking or smoking cessation, Moderately vigorous physical activity at least 30 minutes 3–5 times/week, BMI <25 kg/m^2^, waist circumference <94 cm in men (<90 cm in South-East Asian men) and <80 cm in women, blood pressure <140/90 mmHg (<140/85 mmHg in diabetics), LDL <1.8 mmol/L, LDL <1.4 mmol/L, non-HDL cholesterol <2.2 mmol/L, triglycerides <1.7 mmol/L, and Hba1c (in diabetic patients) <7%. ^b^ Estimates are calculated among diabetic patients. ^c^ 97.5% CI = 0.999.

### Treatment

Most patients were using antiplatelet (92.6%), antihypertensive (94.2%), and lipid-lowering (89.3%) medication. Half of the study participants participated in a cardiac rehabilitation program (50.5%), with important variation by country-income status: participation was 74.5% in HICs, 25.7% in UMICs, and 21.8% in LMICs (Figure 1, **Supplementary Table 1**).

Higher educational level was positively associated with the use of antiplatelet medication and antihypertensives (HICs) and lipid-lowering drugs (HICs and LMICs), but negatively associated with and insulin among diabetics and lipid-lowering drugs in UMICs. Higher education was strongly associated with participation in cardiac rehabilitation in HICs and UMICs (Table 3).

### Sensitivity analysis

Levels of risk factor recording for some variables, such as physical activity, BMI, and blood lipids, were lower among patients whose data was collected through pre-existing sources as compared to interview (**Supplementary Table 3**). Otherwise, no major differences by data collection source were observed in patients’ characteristics, attainment of risk factors, treatment, or the associations between educational level and target attainment or treatment. Similarly, sensitivity analysis by CHD diagnostic category showed no major differences in study outcomes (**Supplementary File 2**).

## Discussion

### Summary of main findings

In our study of 13,884 patients with CHD from 29 countries in Europe, the Middle-East, North- and South America, and Asia, we observed reasonable recording of most risk factors and high use of most medication classes, but poor attainment of risk factor targets and participation in cardiac rehabilitation. Patients with higher education are generally more likely to meet risk factor targets, to receive medical treatment, and to participate in cardiac rehabilitation, although these associations varied in different country income groups.

### Recording

Our findings show that blood pressure and smoking were registered in almost all patients; however, recording for other risk factors such as blood lipids, Hba1c, and BMI were modest, and very low for waist circumference. Previous studies similarly found satisfactory recording of blood pressure in primary care [16,17], and of blood pressure and smoking in secondary prevention [18], reporting incomplete data on other risk factors too. We also observed lower risk factor recording for some variables in data collected from pre-existing sources as compared to interviews, which can be partly explained by the structure of these data sources as some variables are not collected by design. Overall, the low level of recording observed is cause for concern because risk factor recording is a key step for efficient counseling, adaptation to therapy, and follow-up [16,17,18].

### Target attainment

Our results show poor levels of target attainment in secondary prevention, especially for weight-related risk factors and blood lipids. These findings are in line with previous studies [7,18,19,20], and these highlight the need to improve risk factor control in CHD patients.

Patients with higher educational level were generally more likely to meet risk factor targets, although these associations varied by country income group and risk factor. A higher educational level was associated with meeting the target for smoking in all country-income groups, while a negative association was observed for waist circumference and LDL targets in UMICs, and for BMI targets in LMICs.

A positive association between educational level and risk factor target attainment has been reported in previous studies [8,20]. In EUROASPIRE V, CHD patients with primary or secondary education were less likely to meet risk factor targets for most cardiovascular risk factors compared to patients with tertiary education [8]. Higher educational level were associated with achieving physical activity targets in Swedish CHD patients [20], and with having a healthy diet and not smoking in participants of the community-based PURE Study [10].

The differences in risk factor target attainment by educational level shown in our results could be partly attributed to risk factor awareness and health literacy. Patients with a higher educational level are more likely to be aware of their risk factors, measured levels, and targets [8], as well as to have more extensive health literacy [21,22]. Patient’s awareness of their risk factor profile is a key to motivate lifestyle changes, and it is associated with prevention-seeking behaviors and risk factor control [23,24]. Adequate health literacy allows patients to understand health-related information and make informed decisions [25,26]. Knowledge on risk factors has been associated with improved health behaviors [23], and results from a systematic review [25] show that patients with low health literacy have less knowledge on preventive methods and use of preventive health services.

Differences in care provision by public and private centers can contribute to the differential target attainment by educational level. Therefore, we included the private or public organization of participating centers in our models. Most SURF CHD II patients in HICs, with generally strong public healthcare systems and universal health coverage [27], attended public centers regardless of their educational level, whereas most patients with higher education attended private centers in LMICs [**Supplementary Table 2**]. Previous studies conducted in Brazil showed that CHD patients treated in the private system were more likely to meet the physical activity target, and to use and adhere to guideline-recommended medications [28,29] suggesting that patients using private care in such settings may have better access to medications [30], as well as more frequent healthcare utilization [31], and thus a more effective management of risk factors. As provision of services in public and private health systems varies greatly by country, future specific analysis should allow for in-depth research on this topic. Variations in target attainment by country income group (such as in BMI and waist circumference), could further be influenced by the differential ethnic distribution; for example, the majority of participants in LMICs were Asian, while most patients in HICs were white.

Differences in risk factors at baseline by educational level could also play a role in the association between educational level and target attainment. In our data, for example, the proportion of patients who never smoked is similar across educational level groups, while the proportion of patients who quit or were current smokers varies by educational level [**Supplementary Table 2**]. However, changes in risk factors could not be assessed due to the cross-sectional design of the study.

### Medication and cardiac rehabilitation

We observe overall high levels of usage of all medication classes in SURF CHD II, in line with the previous surveys in secondary prevention [18,32]. Patients with secondary or tertiary education were more likely to use antiplatelet, antihypertensive, and lipid-lowering medications in HICs, and lipid-lowering medication and oral hypoglycemics in LMICs, while a more inconsistent pattern was observed in UMICs. Although these differences were significant, in many cases these differences were small; for example, 96.7% vs. 95.2% for antihypertensives, and 94.6% vs 91.9% for lipid-lowering drugs in HICs [**Supplementary Table 1**]. Ohm et al. described higher statin use among higher educated patients [20], while the PURE study described higher medication use among lower educated patients in HICs [10], and the most recent EUROASPIRE survey reported no differences in secondary prevention medication use by educational level [8].

Half of the participants in SURF CHD II reported to have participated in a cardiac rehabilitation program, which is a higher estimate than the one reported in Euroaspire IV [33]. Cardiac rehabilitation has proven to be effective in reducing morbidity and mortality risk in coronary patients, and a comprehensive cardiac rehabilitation program has class 1 A recommendation by clinical guidelines [5,34]. Attendance to cardiac rehabilitation was remarkably lower in UMICs (25.7%) and LMICs (21.8%) compared to HICs (74.5%).

Patients with primary educational level were less likely to participate in cardiac rehabilitation in HICs and UMICs. Accordingly, previous studies in Europe and the US have shown lower referral rates for cardiac rehabilitation [35], and lower participation attendance in patients with a lower educational level compared to those with higher education. [33,36]. Barriers to cardiac rehabilitation, like lack of availability or access to programs, low awareness on the program benefits, large distances to health centers, out-of-pocket payments, and disadvantages and costs caused by absence from work [20,37], may impact patients with a lower educational level disproportionately. Cardiac rehabilitation programs are available only in 54.7% of countries [38], and, especially in UMICs and LMICs, there are financial barriers associated with coverage for cardiac rehabilitation [39,40].

Our results highlight that even with high levels of medication use, risk factor target attainment remained poor. Further research on the use of drug combinations, dose adequacy, and adherence, might help to clarify the difficulties controlling risk factors. The fact that higher educated participants were generally more likely to use medication and to attend cardiac rehabilitation could partly contribute to the higher levels of risk factor target attainment among patients with secondary or tertiary education.

### Implications and future research

Our results emphasize the importance of addressing barriers to risk factor target attainment and cardiac rehabilitation that are specific to people with lower educational levels. This could be adapted communication strategies, intensive and personalized follow-up to improve target attainment, and promotion of access to affordable and (partly) remote cardiac rehabilitation programs.

One of the main findings of our study is that the associations between educational level and risk factor target attainment and treatment are heterogeneous. Therefore, future studies should investigate local circumstances that hinder risk factor target attainment and treatment in daily practice, with attention to patients’ educational attainment. The resulting insights may support the design of efficient preventive strategies at regional, country, and center level.

### Strengths and limitations

Our study is among the first to investigate risk factor recording, target attainment, and treatment in secondary prevention by educational level and country income group in a clinical setting. Research on risk factor recording is scarce, especially for secondary prevention, and we present results for registration of risk factor information in daily practice. The simplicity of the SURF CHD II audit allows registration of the most relevant information of risk factors, while requiring little time and few financial resources. This also facilitates participation of smaller units and low-resource areas, which often have been underrepresented in research, despite high levels of cardiovascular risk [41]. Therefore, SURF CHD provides real-world evidence on secondary prevention globally, and its large sample size allows for context specific analysis. SURF CHD II provides a useful tool for health centers of any level to assess secondary prevention outcomes in their specific context and apply and evaluate tailor-made prevention strategies.

This study has some limitations. First, centers were not randomly selected, although diversity among the included centers was promoted. Second, health professionals were instructed to only register information collected during routine visits, but it is possible that some additional measurements were performed. These factors may have resulted in some overestimation of the risk factor recording, target attainment, and treatment levels in our results. Additionally, the high level of missing values for BMI, waist circumference, and lipid measurements, may influence the recording and target attainment estimates. As educational level was not registered in some centers, we were, unfortunately, not able to include these participants in the main analysis. Although the simplicity of SURF CHD II is one of its main strengths, it inevitably limits the information that can be collected for study participants, including data that might have provided more insights into the results, like in-depth information on risk factors, sex-specific risk factors, statin intensity, adherence to medication, or time since index event.

### Conclusion

The SURF CHD II study conducted in 13,884 CHD patients from 29 HICs, UMICs, and LMICs provides global, real-world evidence on secondary prevention of CHD. SURF CHD II shows poor attainment of risk factor targets and participation in cardiac rehabilitation, highlighting the urge for improvement in secondary prevention of CHD in daily practice.

The association between educational level and risk factor target attainment is heterogeneous and complex. Further research into health inequalities on secondary prevention outcomes in different contexts might support the identification of barriers to secondary prevention and the application of more effective preventive strategies, which are most needed.

## Data Accessibility Statement

Data is available upon reasonable request to co-authors.

## Additional Files

The additional files for this article can be found as follows:

10.5334/gh.1340.s1 Supplementary File 1. Supplementary Figure 1, Supplementary Tables 1 to 3.

10.5334/gh.1340.s2 Supplementary File 1. Sensitivity Analysis.

## Figures and Tables

**Table:** 


SURF CHD Collaborators	

Marina Joseph, Yanina Castillo Costa	Socieded Argentina de Cardiología, Buenos Aires

Nicolás Esteybar Enrique	Counsel of Cardioecology and Healthy Habits Sociedad Argentina de Cardiología, Mar del Plata, Argentina

Rahima Gabulova	Azerbaijan Medical University, Educational Therapeutic Clinic, Baku, Azerbaijan

Mahluga Isaveva	Scientific Research Institute for Cardiology, Baku, Azerbaijan

Farid Alivev	Baku Health Centre, Baku, Azerbaijan

Uzeyir Rahimov	Baku Medical Plaza, Baku, Azerbaijan

Galib Imanov	Azerbaijan Medical University, Educational Surgical Clinic, Baku, Azerbaijan

Firdovsi Ibrahimov	Central Clinic Hospital, Baku, Azerbaijan

Zarbaliyeva Naila	Ganja City United Hospital, Ganja, Azerbaijan

Rashad Abasov	ER Medical, Khachmaz, Azerbaijan

Paul Dendale, Anre Jassen	Jessa Hospital-Hartcentrum, Hasselt, Belgium

Johan De Sutter	AZ Maria Middelares Cardiologie, Gent, Belgium

Sofie Cuypers	Hartcentrum OLV ziekenhuis, Aalst, Belgium

Dalton Precoma	Sociedade Hospitalar Angelina Caron, Campina Grande do Sul, Brazil

Luiz Ritt	Escola Bahiana de Medicina e Saúde Pública, Salvador, Brazil

Mario Claudio Soares Sturzeneker	Wallace Thadeu de Mello e Silva Regional University Hospital, Brazil

Conrado Roberto Hoffmann Filho	Street Blumenau, 294 Centro, Joinville SC, Brazil

Maria Teresa Lira	Hospital Clínico Fuerza Aérea de Chile, Santiago, Chile

Michal Varablik, Eva Tumova	Centre of Preventive Cardiology, 3th Department of internal medicine, 1st Faculty of Medicine and General Teaching Hospital, Prague, Czech Republic

Jaromir Ozana	Department of Exercise Medicine and Cardiovascular Rehabilitation, University Hospital Olomouc, Czech Republic

Ann Bovin	Danish Society of Cardiology, Copenhagen, Denmark

Margus Viigimaa	North Estonia Medical Centre, Tallinn University of Technology, Tallin, Estonia

Konstantinos Tsioufis, Ageliki Laina	Cardiology Department, Hippokration General Hospital, Athens, Greece

Zacharoulis Achilles, Fotios Toulgaridis	General Hospital of Athens “Evangelismos”, Athens, Greece

Elias Sanidas	LAIKO General Hospital, Athens, Greece

Zeljko Reiner	Polish Mother’s Memorial Hospital Research Institute, Lodz, Poland & University Hospital Center, Zagreb, Croatia

Marijana Gulin, Antonijo BejúiĆ	County Hospital Sibenik, Croatia

Darko Duplančić	University Hospital, Split, Croatia

Jozica Šikić	University Hospital Sveti Duh, Croatia

Eszter Szabados	Ist Department of Medicine, Division of Preventive Cardiology and Rehabilitation, University of Pécs, Medical School, Pécs, Hungary

Badai Bhatara Tiksnadi	Hasan Sadikin General Hospital, Bandung, West Java, Indonesia

Bill McEvoy	University Hospital Galway, Galway, Ireland

Anne Reynolds, David Moore	Tallaght University, Hospital Cardiology clinic, Dublin, Ireland

Declan Spelman	South Tipperary General Hospital, Tipperary, Ireland

Raman Puri, Rashmi Nanda	Cardiac Care Centre, New Delhi, India

Nagaraj Desai	Namana Medical Centre, Bengaluru, India

Prabhakar Dorairaj	Ashwin Clinic, Annanagar, India

S. S. Iyengar, Sumitra Lakshmana	Manipal Hospital, Bangalore, India

Ajay Kumar Pandey	Galaxy Hospital, Mahmoorganj, India

Akshyaya Pradhan	King George’s Medical University, Lucknow, India

Kunal Mahajan	Himachal Heart Institute, Chhanwari, India

Pompilio Faggiano	Ospedali Riuniti, Brescia, Italy

P. Zarcone	Policlinico P. Giccone, Palermo, Italy

Maurizio G Abrignani	S Antonio Abate Hospital of Trapani, O.U. of Cardiology, Trapani, Italy

Makhanov Daniyar	Central Clinical Hospital, Almaty, Kazakhstan

Kairat Davletov	Asfendiyarov Kazakh National Medical University, Almaty, Republic of Kazakhstan

Kuatbek Toleubekov, Olga Visternichan	Cardiorehabilitation Center “Tulpar”, Karaganda Medical University, Karaganda, Kazakhstan

Alibek Mereke	City Policlinic № 32, Al Farabi Kazakh National University, Almaty, Kazakhstan

Anar Dushpanova	City Policlinic № 32, Al Farabi Kazakh National University, Almaty, Kazakhstan; Scuola Superiore Sant’Anna, Pisa, Italy

Bekbolat Zholdin, Zhanat Timirbayev	Medical Center of West Kazakhstan Marat Ospanov Medical University, Zhanakonys, Kazakhstan

Gulmira Derbissalina	Astana Medical University, University hospital, Astana, Kazakhstan

Daulet K. Aldyngurov	Department of Science and Human resource, Ministry of Healthcare, Kazakhstan

Ayan Myssayev	Cardiological Center of Pavlodar region, Department of Science and Human resourse, Ministry of Healthcare, Kazakhstan

Alma Nurtazina	Cardiological Center of Pavlodar region, Semey Medical University, Semey, Kazakhstan

Zhanna Romanova, Sundetgali Kalmakhanov	City polyclinic № 5, Al Farabi Kazakh National University, Almaty, Kazakhstan

Iveta Mintale	Latvian Center of Cardiology, Riga, Latvia

Omar Msalam	Libyan Cardiac Society, Tripoli, Libya

Emhemed Ehemmali	Misrata Heart & Cardiovascular Center, Misrata, Libya

Alami Mohamed	Cabinet Cardiologie ALAMI, Casablanca, Morocco

Najat Mouine	Cardiology Centre, Mohammed V Military Hospital, Rabat, Morocco

Tazi Mezalek Amale	Hopital Universitaire Cheikh Zaid, Rabat, Morocco

Aida Soufiani, Zineb Agoumy, Houda Bachri, Imad Massri	Ligue Nationale de cardiologie, Rabat, Morocco

Irena Mitevska	University Cardiology Clinic, Skopje, North Macedonia

Saskia Haitjema, Mark de Groot	Utrecht Patient Oriented Database (UPOD), Central Diagnostic Laboratory, Division of Laboratory, Pharmacy, and Biogenetics, University Medical Center Utrecht, Utrecht University, Utrecht

Ana Abreu	Hospital Universitário Santa Maria, IMPSP, ISAMB, Faculty of Medicine of University of Lisbon, Lisbon

Svetlana Mosterou, Dan Gaita	Institute for Cardiovascular Diseases, Timisoara, Romania

Nana Pogosova	National Medical Research Center of Cardiology, Preventive Cardiology Laboratory, Moscow, Russia

Marat V. Ezhov	Federal State Budget Institution, National Cardiology Research Centre of Ministry of Healthcare of Russian Federation, Moscow, Russia

Abdulhalim Kinsara	Ministry of National Guard health affairs. King Saud bin Abdulaziz University for Health Sciences, COM-WR. King Abdullah International Research Center, Jeddah, Saudi Arabia

Ivana Burazor	Cardiology, Institute for rehabilitation; Cardiology, Institute for rehabilitation, and University of Belgrade – Faculty of Medicine, Institute for Cardiovascular Diseases ‘Dedinje’, Belgrade, Serbia

Vojislav Giga	Cardiology Clinic, Clinical Center of Serbia, Belgrade, Serbia

Hector Bueno	Hospital 12 de Octubre, Madrid, and Centro Nacional de Investigaciones Cardiovasculares, Madrid, Spain

Regina Dalmau	University Hospital la Paz, Madrid, Spain

Ana García-Barrios	Hospital General Universitario Dr. Balmis, Alicante, Spain

Jose Antonio Alarcon Duque, Joana Reparaz Mendinueta	Hospital Universitario Donostia/OSI Donostialdea, Donostia, Spain

Meral Kayikcioglu	Ege University Medical School Cardiology Department, Ege University Tip Fak Kardivoloii AD Bornova Izmir, Turkey

Lale Tokgozoglu	Hacettepe University, Department of Cardiology, Ankara, Turkey

Emre Aslanger	Basaksehir Pine and Sakura City Hospital, Istanbul, Turkey

Ayca Turer Cabbar	Yeditepe Univerisity Hospital, Istanbul, Turkey

Samuel Kim, Caleb Self, Dolores Reynolds, Sabrina Rose, Pretti Singh	Weil Cornell Medicine, New York, United States

