# Supplementary material for: A Global Perspective on Cardiovascular Risk Factors by Educational Level in CHD Patients: SURF CHD II
Source: Glob Heart. 2024 Jul 16;19(1):60. doi: 10.5334/gh.1340 (PMC11259115; doi:10.5334/gh.1340)
Supplement: Supplementary File 1. — Supplementary Figure 1, Supplementary Tables 1 to 3. [file gh-19-1-1340-s1.pdf]

SUPPLEMENTARY MATERIAL

Supplementary Figure 1

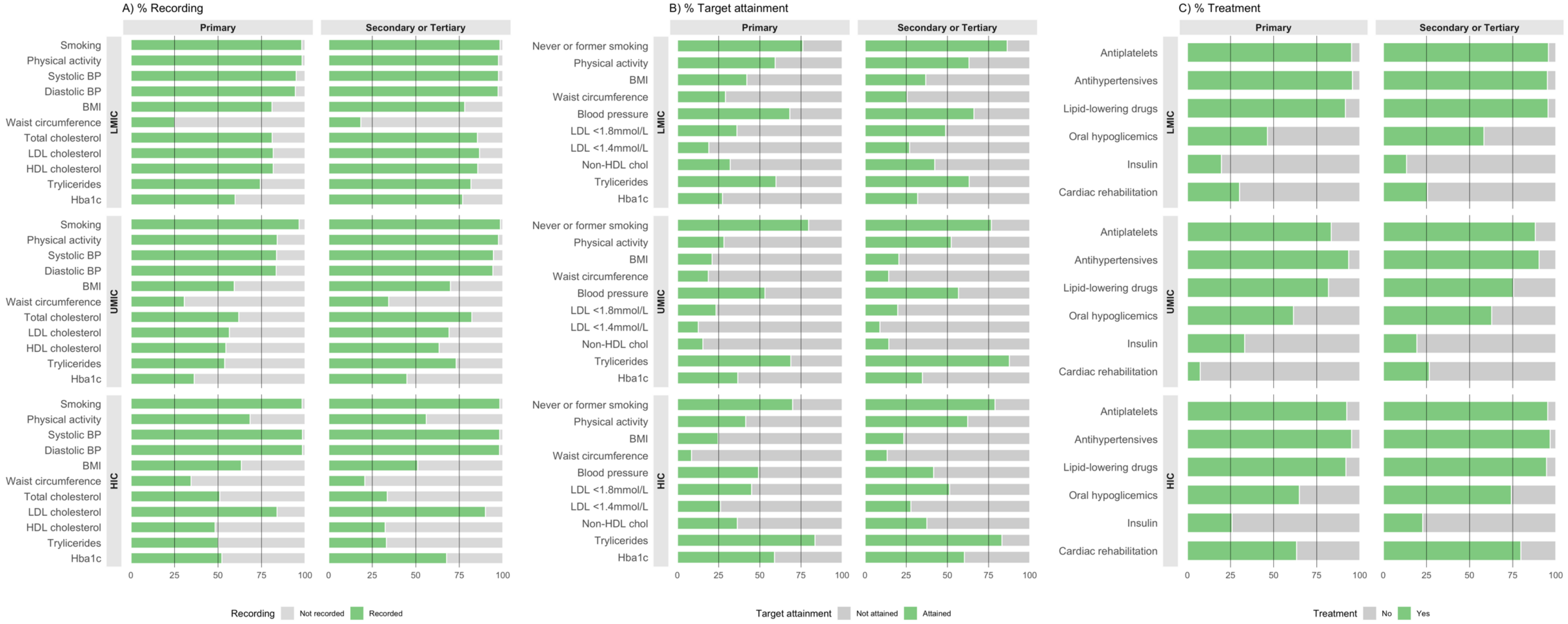

Supplementary Table 1. Risk factor recording, attainment and treatment by country income group (%).

	LMIC (N=2645)	UMIC (N=3777)	HIC (N =7462)	Total (N=13884)
**Recording**				
Smoking history				
No	44 (1.7)	143 (3.8)	419 (5.6)	606 (4.4)
Yes	2601 (98.3)	3634 (96.2)	7043 (94.4)	13278 (95.6)
Physical actiivity				
No	67 (2.5)	585 (15.5)	3698 (49.6)	4350 (31.3)
Yes	2578 (97.5)	3192 (84.5)	3764 (50.4)	9534 (68.7)
Systolic blood pressure				
No	80 (3.0)	337 (8.9)	557 (7.5)	974 (7.0)
Yes	2565 (97.0)	3440 (91.1)	6905 (92.5)	12910 (93.0)
Diastolic blood pressure				
No	88 (3.3)	345 (9.1)	560 (7.5)	993 (7.2)
Yes	2557 (96.7)	3432 (90.9)	6902 (92.5)	12891 (92.8)
BMI				
No	608 (23.0)	1365 (36.1)	3710 (49.7)	5683 (40.9)
Yes	2037 (77.0)	2412 (63.9)	3752 (50.3)	8201 (59.1)
Waist circumference				
No	2116 (80.0)	2723 (72.1)	5968 (80.0)	10807 (77.8)
Yes	529 (20.0)	1054 (27.9)	1494 (20.0)	3077 (22.2)
Total cholesterol				
No	436 (16.5)	945 (25.0)	4456 (59.7)	5837 (42.0)
Yes	2209 (83.5)	2832 (75.0)	3006 (40.3)	8047 (58.0)
LDL cholesterol				
No	409 (15.5)	1304 (34.5)	1271 (17.0)	2984 (21.5)
Yes	2236 (84.5)	2473 (65.5)	6191 (83.0)	10900 (78.5)
HDL cholesterol				
No	429 (16.2)	1531 (40.5)	4565 (61.2)	6525 (47.0)
Yes	2216 (83.8)	2246 (59.5)	2897 (38.8)	7359 (53.0)
Triglycerides				
No	550 (20.8)	1236 (32.7)	4503 (60.3)	6289 (45.3)
Yes	2095 (79.2)	2541 (67.3)	2959 (39.7)	7595 (54.7)
Hba1c (among diabetics)[a]				
No	309 (27.2)	793 (61.3)	493 (43.7)	1595 (44.8)
Yes	827 (72.8)	501 (38.7)	636 (56.3)	1964 (55.2)
**Target attainment[b]**				
Smoking target (Never or former smoking)				
No	423 (16.3)	861 (23.7)	1541 (21.9)	2825 (21.3)
Yes	2178 (83.7)	2773 (76.3)	5502 (78.1)	10453 (78.7)
Missing	44 (1.7)	143 (3.8)	419 (5.6)	606 (4.4)
Physical activity				
No	979 (38.0)	1690 (52.9)	1640 (43.6)	4309 (45.2)
Yes	1599 (62.0)	1502 (47.1)	2124 (56.4)	5225 (54.8)
Missing	67 (2.5)	585 (15.5)	3698 (49.6)	4350 (31.3)
BMI				

No	1241 (61.4)	1906 (79.1)	3123 (75.0)	6270 (73.0)
Yes	779 (38.6)	504 (20.9)	1041 (25.0)	2324 (27.0)
Missing	625 (23.6)	1367 (36.2)	3298 (44.2)	5290 (38.1)
Waist circumference				
No	383 (72.4)	897 (85.2)	1307 (87.5)	2587 (84.1)
Yes	146 (27.6)	156 (14.8)	186 (12.5)	488 (15.9)
Missing	2116 (80.0)	2724 (72.1)	5969 (80.0)	10809 (77.9)
Blood pressure				
No	851 (33.2)	1550 (45.1)	2385 (53.4)	4786 (45.7)
Yes	1711 (66.8)	1886 (54.9)	2084 (46.6)	5681 (54.3)
Missing	83 (3.1)	341 (9.0)	2993 (40.1)	3417 (24.6)
LDL <1.8mmol/L				
No	1234 (55.2)	1985 (80.3)	3238 (52.3)	6457 (59.2)
Yes	1002 (44.8)	488 (19.7)	2953 (47.7)	4443 (40.8)
Missing	409 (15.5)	1304 (34.5)	1271 (17.0)	2984 (21.5)
LDL 1.4mmol/L				
No	1686 (75.4)	2260 (91.4)	4686 (75.7)	8632 (79.2)
Yes	550 (24.6)	213 (8.6)	1505 (24.3)	2268 (20.8)
Missing	409 (15.5)	1304 (34.5)	1271 (17.0)	2984 (21.5)
Non-HDL target				
No	1333 (61.1)	1864 (84.8)	1714 (61.4)	4911 (68.5)
Yes	849 (38.9)	333 (15.2)	1077 (38.6)	2259 (31.5)
Missing	463 (17.5)	1580 (41.8)	4671 (62.6)	6714 (48.4)
Triglycerides				
No	800 (38.2)	982 (38.6)	880 (29.7)	2662 (35.0)
Yes	1295 (61.8)	1559 (61.4)	2079 (70.3)	4933 (65.0)
Missing	550 (20.8)	1236 (32.7)	4503 (60.3)	6289 (45.3)
Hba1c[a]				
No	569 (68.8)	323 (64.5)	277 (43.6)	1169 (59.5)
Yes	258 (31.2)	178 (35.5)	359 (56.4)	795 (40.5)
Missing	309 (27.2)	793 (61.3)	493 (43.7)	1595 (44.8)
**Treatment**				
Antiplatelets				
No	114 (4.3)	456 (12.1)	424 (6.0)	994 (7.4)
Yes	2531 (95.7)	3321 (87.9)	6630 (94.0)	12482 (92.6)
Missing	0 (0)	0 (0)	408 (5.5)	408 (2.9)
Anithypertensives				
No	124 (4.7)	359 (9.5)	230 (4.0)	713 (5.8)
Yes	2521 (95.3)	3418 (90.5)	5575 (96.0)	11514 (94.2)
Missing	0 (0)	0 (0)	1657 (22.2)	1657 (11.9)
Lipid-lowering				
No	147 (5.6)	860 (22.8)	435 (6.2)	1442 (10.7)
Yes	2498 (94.4)	2917 (77.2)	6604 (93.8)	12019 (89.3)
Missing	0 (0)	0 (0)	423 (5.7)	423 (3.0)
Oral hypoglicemics[a]				
No	506 (44.5)	515 (39.8)	360 (31.9)	1381 (38.8)
Yes	630 (55.5)	779 (60.2)	769 (68.1)	2178 (61.2)
Insulin [a]				
No	953 (83.9)	1011 (78.1)	806 (71.4)	2770 (77.8)
Yes	183 (16.1)	283 (21.9)	323 (28.6)	789 (22.2)
Cardiac rehabilitation				
No	1961 (74.3)	2684 (78.2)	1737 (25.5)	6382 (49.5)
Yes	677 (25.7)	749 (21.8)	5076 (74.5)	6502 (50.5)
Missing	7 (0.3)	344 (9.1)	649 (8.7)	1000 (7.2)

Footnote: Results are indicated in percentages unless indicated. [a] Percentages are provided among diabetic patients. Number of diabetic patients was 1136 (LMICs), 1294 (UMICs), and 1129 (HICs). [b] Risk factor targets are defined as: no smoking or smoking cessation, Moderately vigorous physical activity at least 30 minutes 3-5 times/week, BMI <25 kg/m^2, waist circumference <94 cm in men (<90cm in South-East Asian men) and <80 cm in women, blood pressure <140/90 mmHg (<140/85 mmHg in diabetics), LDL <1.8mmol/L, LDL <1.4mmol/L, non-HDL cholesterol <2.2 mmol/L, triglycerides <1.7 mmol/L, and Hba1c (in diabetic patients) <7%. HICs: high-income counties, UMICs: upper-middle-income countries, LMICs: lower-middle income countries.

Supplementary Table 2: Characteristics of the study population by country income group and educational level.

	LMIC		UMIC		HIC	
	Primary (N=630)	Secondary or Tertiary (N=1914)	Primary (N=506)	Secondary or Tertiary (N=2503)	Primary (N=921)	Secondary or Tertiary (N=4601)
**Participants's cahartceristics**						
Centre type						
Private	238 (37.8)	1243 (64.9)	183 (36.2)	442 (17.7)	7 (0.8)	139 (3.2)
Public	392 (62.2)	671 (35.1)	323 (63.8)	2061 (82.3)	830 (99.2)	4261 (96.8)
Missing	0 (0)	0 (0)	0 (0)	0 (0)	84 (9.1)	201 (4.4)
Age (mean (SD))	63.5 (11.6)	60.5 (11.6)	66.6 (10.7)	62.2 (10.4)	68.1 (11.8)	65.7 (10.6)
Sex						
Female	198 (31.4)	279 (14.6)	207 (40.9)	687 (27.4)	314 (34.1)	970 (21.1)
Male	432 (68.6)	1635 (85.4)	299 (59.1)	1816 (72.6)	607 (65.9)	3631 (78.9)
Ethnic group						
Arab	93 (14.8)	205 (10.7)	35 (6.9)	63 (2.5)	19 (2.1)	25 (0.5)
Asian	533 (84.6)	1700 (88.8)	25 (5.0)	440 (17.6)	1 (0.1)	9 (0.2)
Black	1 (0.2)	5 (0.3)	12 (2.4)	3 (0.1)	1 (0.1)	1 (0.0)
Mixed	1 (0.2)	2 (0.1)	28 (5.5)	7 (0.3)	1 (0.1)	2 (0.0)
White	2 (0.3)	2 (0.1)	405 (80.2)	1987 (79.4)	425 (46.2)	1481 (32.2)
Other - Turkik	0 (0)	0 (0)	0 (0)	2 (0.1)	0 (0)	0 (0)
Not registered in original dataset	0 (0)	0 (0)	0 (0)	0 (0)	473 (51.4)	3081 (67.0)
Missing	0 (0)	0 (0)	1 (0.2)	1 (0.0)	1 (0.1)	2 (0.0)
Educational level						
Primary school	630 (100)	0 (0)	506 (100)	0 (0)	921 (100)	0 (0)
Secondary school	0 (0)	964 (50.4)	0 (0)	1403 (56.1)	0 (0)	1451 (31.5)
Tertiary education	0 (0)	950 (49.6)	0 (0)	1100 (43.9)	0 (0)	3150 (68.5)
Index event						
CABG						
No	558 (88.6)	1574 (82.2)	398 (78.7)	2032 (81.2)	537 (82.0)	2248 (83.7)
Yes	72 (11.4)	340 (17.8)	108 (21.3)	471 (18.8)	118 (18.0)	437 (16.3)
Missing	0 (0)	0 (0)	0 (0)	0 (0)	266 (28.9)	1916 (41.6)
PCI						
No	374 (59.4)	962 (50.3)	206 (40.7)	950 (38.0)	259 (39.5)	1176 (43.8)
Yes	256 (40.6)	952 (49.7)	300 (59.3)	1553 (62.0)	396 (60.5)	1509 (56.2)
Missing	0 (0)	0 (0)	0 (0)	0 (0)	266 (28.9)	1916 (41.6)
Acute coronary syndrome						
No	355 (56.3)	1230 (64.3)	279 (55.1)	1545 (61.7)	359 (39.0)	1825 (39.7)
Yes	275 (43.7)	684 (35.7)	227 (44.9)	958 (38.3)	562 (61.0)	2776 (60.3)

Stable angina pectoris						
No	405 (64.3)	1299 (67.9)	320 (63.2)	1556 (62.2)	707 (76.8)	3351 (72.8)
Yes	225 (35.7)	615 (32.1)	186 (36.8)	947 (37.8)	214 (23.2)	1250 (27.2)
**Risk factor history**						
Smoking history						
Current smoker	148 (23.9)	258 (13.7)	100 (20.4)	576 (23.3)	272 (30.1)	953 (21.1)
Ex-smoker	73 (11.8)	349 (18.5)	175 (35.8)	814 (33.0)	343 (37.9)	2120 (46.9)
Never smoked	397 (64.2)	1276 (67.8)	214 (43.8)	1077 (43.7)	290 (32.0)	1452 (32.1)
Missing	12 (1.9)	31 (1.6)	17 (3.4)	36 (1.4)	16 (1.7)	76 (1.7)
Physical activity						
Less than moderate	252 (40.1)	689 (36.0)	305 (60.3)	1163 (46.5)	369 (57.1)	972 (36.7)
Moderate (moderately vigorous) 30 min 3-5 times/week	293 (46.6)	929 (48.5)	80 (15.8)	863 (34.5)	183 (28.3)	1078 (40.7)
More than moderate	74 (11.8)	248 (13.0)	40 (7.9)	414 (16.5)	78 (12.1)	527 (19.9)
Missing	10 (1.6)	48 (2.5)	81 (16.0)	63 (2.5)	16 (2.5)	69 (2.6)
Previous history of hypertension						
No	340 (54.0)	889 (46.4)	92 (18.2)	427 (17.1)	219 (32.3)	1064 (37.1)
Yes	290 (46.0)	1025 (53.6)	414 (81.8)	2076 (82.9)	460 (67.7)	1802 (62.9)
Missing	0 (0)	0 (0)	0 (0)	0 (0)	242 (26.3)	1735 (37.7)
Previous history of dyslipidemia						
No	460 (73.0)	880 (46.0)	210 (41.5)	1055 (42.1)	261 (39.7)	947 (34.0)
Yes	170 (27.0)	1034 (54.0)	296 (58.5)	1448 (57.9)	396 (60.3)	1837 (66.0)
Missing	0 (0)	0 (0)	0 (0)	0 (0)	264 (28.7)	1817 (39.5)
Previous history of diabetes						
No	391 (62.1)	1058 (55.3)	280 (55.3)	1659 (66.3)	327 (35.5)	1248 (27.1)
Yes	239 (37.9)	856 (44.7)	226 (44.7)	844 (33.7)	205 (22.3)	473 (10.3)
Not registered in original dataset	0 (0)	0 (0)	0 (0)	0 (0)	389 (42.2)	2880 (62.6)
**Risk factor measurements and laboratory values**						
Systolic BP (mmHg) (mean (SD))	128 (22.1)	129 (19.5)	135 (21.0)	133 (19.4)	132 (19.0)	132 (17.1)
Missing	32 (5.1)	47 (2.5)	83 (16.4)	135 (5.4)	14 (1.5)	85 (1.8)
Diastolic BP (mmHg) (mean (SD))	74.9 (11.6)	76.2 (11.1)	78.1 (12.6)	80.7 (10.7)	76.0 (11.6)	77.8 (10.2)
Missing	35 (5.6)	52 (2.7)	84 (16.6)	142 (5.7)	14 (1.5)	88 (1.9)
BMI (kg/m^2) (mean (SD))	26.2 (4.44)	26.6 (4.47)	28.6 (4.71)	28.6 (4.70)	28.4 (5.56)	28.4 (4.90)
Missing	129 (20.5)	427 (22.3)	206 (40.7)	755 (30.2)	337 (36.6)	2254 (49.0)
Waist circumference (cm) (mean (SD))	84.9 (22.3)	92.1 (12.5)	96.8 (12.9)	99.5 (12.7)	103 (12.3)	103 (13.6)
Missing	581 (92.2)	1705 (89.1)	365 (72.1)	1679 (67.1)	627 (68.1)	3672 (79.8)

Total cholesterol (mmol/L) (mean (SD))	4.02 (1.25)	3.79 (1.25)	4.69 (1.60)	4.73 (1.36)	4.01 (1.66)	4.04 (1.55)
Missing	119 (18.9)	280 (14.6)	193 (38.1)	446 (17.8)	451 (49.0)	3056 (66.4)
LDL (mmol/L) (mean (SD))	2.28 (1.05)	2.09 (1.08)	2.67 (1.37)	2.82 (1.20)	2.22 (1.21)	2.07 (1.04)
Missing	115 (18.3)	257 (13.4)	220 (43.5)	773 (30.9)	148 (16.1)	460 (10.0)
HDL (mmol/L) (mean (SD))	1.15 (0.37)	1.10 (0.33)	1.13 (0.35)	1.19 (0.40)	1.20 (0.47)	1.21 (0.50)
Missing	115 (18.3)	276 (14.4)	230 (45.5)	916 (36.6)	476 (51.7)	3110 (67.6)
Triglycerides (mmol/L) (mean (SD))	3.81 (1.71)	3.59 (1.63)	3.41 (2.18)	2.20 (1.58)	2.60 (1.53)	2.67 (1.82)
Missing	164 (26.0)	362 (18.9)	237 (46.8)	680 (27.2)	462 (50.2)	3080 (66.9)
Glucose (mmol/L) (mean (SD))	7.62 (2.99)	7.21 (2.70)	7.14 (3.04)	6.61 (2.46)	6.60 (2.22)	6.28 (1.84)
Missing	168 (26.7)	486 (25.4)	206 (40.7)	624 (24.9)	475 (51.6)	3309 (71.9)
Hba1c (%) (mean (SD))[a]	8.40 (1.99)	7.87 (1.62)	7.88 (1.89)	7.68 (1.61)	12.0 (16.2)[c]	7.78 (9.67)
Missing	96 (40.2)	198 (23.1)	145 (64.2)	466 (55.2)	104 (50.7)	186 (39.3)
**Risk factor recording**						
Smoking history						
No	12 (1.9)	31 (1.6)	17 (3.4)	36 (1.4)	16 (1.7)	76 (1.7)
Yes	618 (98.1)	1883 (98.4)	489 (96.6)	2467 (98.6)	905 (98.3)	4525 (98.3)
Physical activity						
No	11 (1.7)	48 (2.5)	81 (16.0)	63 (2.5)	291 (31.6)	2024 (44.0)
Yes	619 (98.3)	1866 (97.5)	425 (84.0)	2440 (97.5)	630 (68.4)	2577 (56.0)
Systolic BP						
No	32 (5.1)	47 (2.5)	83 (16.4)	135 (5.4)	14 (1.5)	85 (1.8)
Yes	598 (94.9)	1867 (97.5)	423 (83.6)	2368 (94.6)	907 (98.5)	4516 (98.2)
Diastolic BP						
No	35 (5.6)	52 (2.7)	84 (16.6)	142 (5.7)	14 (1.5)	88 (1.9)
Yes	595 (94.4)	1862 (97.3)	422 (83.4)	2361 (94.3)	907 (98.5)	4513 (98.1)
BMI						
No	120 (19.0)	419 (21.9)	206 (40.7)	753 (30.1)	337 (36.6)	2249 (48.9)
Yes	510 (81.0)	1495 (78.1)	300 (59.3)	1750 (69.9)	584 (63.4)	2352 (51.1)
Waist circumference						
No	472 (74.9)	1561 (81.6)	351 (69.4)	1641 (65.6)	603 (65.5)	3647 (79.3)
Yes	158 (25.1)	353 (18.4)	155 (30.6)	862 (34.4)	318 (34.5)	954 (20.7)
Total cholesterol						
No	119 (18.9)	280 (14.6)	193 (38.1)	446 (17.8)	453 (49.2)	3058 (66.5)
Yes	511 (81.1)	1634 (85.4)	313 (61.9)	2057 (82.2)	468 (50.8)	1543 (33.5)
LDL cholesterol						
No	115 (18.3)	257 (13.4)	220 (43.5)	773 (30.9)	148 (16.1)	466 (10.1)

Yes	515 (81.7)	1657 (86.6)	286 (56.5)	1730 (69.1)	773 (83.9)	4135 (89.9)
HDL cholesterol						
No	115 (18.3)	276 (14.4)	230 (45.5)	916 (36.6)	476 (51.7)	3110 (67.6)
Yes	515 (81.7)	1638 (85.6)	276 (54.5)	1587 (63.4)	445 (48.3)	1491 (32.4)
Triglycerides						
No	162 (25.7)	350 (18.3)	234 (46.2)	671 (26.8)	460 (49.9)	3077 (66.9)
Yes	468 (74.3)	1564 (81.7)	272 (53.8)	1832 (73.2)	461 (50.1)	1524 (33.1)
Hba1c [a]						
No	96 (40.2)	198 (23.1)	144 (63.7)	465 (55.1)	98 (47.8)	153 (32.3)
Yes	143 (59.8)	658 (76.9)	82 (36.3)	379 (44.9)	107 (52.2)	320 (67.7)
**Risk factor target attainment[b]**						
Smoking target (Never or former smoking)						
No	148 (23.9)	258 (13.7)	100 (20.4)	576 (23.3)	272 (30.1)	953 (21.1)
Yes	470 (76.1)	1625 (86.3)	389 (79.6)	1891 (76.7)	633 (69.9)	3572 (78.9)
Missing	12 (1.9)	31 (1.6)	17 (3.4)	36 (1.4)	16 (1.7)	76 (1.7)
Physical activity						
No	252 (40.7)	689 (36.9)	305 (71.8)	1163 (47.7)	369 (58.6)	972 (37.7)
Yes	367 (59.3)	1177 (63.1)	120 (28.2)	1277 (52.3)	261 (41.4)	1605 (62.3)
Missing	11 (1.7)	48 (2.5)	81 (16.0)	63 (2.5)	291 (31.6)	2024 (44.0)
BMI						
No	290 (57.9)	940 (63.2)	237 (79.0)	1389 (79.5)	440 (75.3)	1796 (76.5)
Yes	211 (42.1)	547 (36.8)	63 (21.0)	359 (20.5)	144 (24.7)	551 (23.5)
Missing	129 (20.5)	427 (22.3)	206 (40.7)	755 (30.2)	337 (36.6)	2254 (49.0)
Waist circumference						
No	112 (70.9)	263 (74.5)	126 (81.3)	739 (85.7)	290 (91.5)	826 (86.6)
Yes	46 (29.1)	90 (25.5)	29 (18.7)	123 (14.3)	27 (8.5)	128 (13.4)
Missing	472 (74.9)	1561 (81.6)	351 (69.4)	1641 (65.6)	604 (65.6)	3647 (79.3)
Blood pressure						
No	190 (31.8)	633 (33.9)	198 (46.9)	1025 (43.3)	335 (36.9)	1490 (33.0)
Yes	407 (68.2)	1232 (66.1)	224 (53.1)	1340 (56.7)	572 (63.1)	3023 (67.0)
Missing	33 (5.2)	49 (2.6)	84 (16.6)	138 (5.5)	14 (1.5)	88 (1.9)
LDL <1.8 mmol/L						
No	329 (63.9)	847 (51.1)	219 (76.6)	1400 (80.9)	448 (58.0)	2177 (52.6)
Yes	186 (36.1)	810 (48.9)	67 (23.4)	330 (19.1)	325 (42.0)	1958 (47.4)
Missing	115 (18.3)	257 (13.4)	220 (43.5)	773 (30.9)	148 (16.1)	466 (10.1)
LDL <1.4mmol/L						
No	417 (81.0)	1210 (73.0)	253 (88.5)	1584 (91.6)	592 (76.6)	3177 (76.8)
Yes	98 (19.0)	447 (27.0)	33 (11.5)	146 (8.4)	181 (23.4)	958 (23.2)
Missing	115 (18.3)	257 (13.4)	220 (43.5)	773 (30.9)	148 (16.1)	466 (10.1)

Non-HDL target						
No	346 (68.0)	930 (57.8)	226 (84.6)	1337 (85.5)	276 (63.4)	878 (62.4)
Yes	163 (32.0)	680 (42.2)	41 (15.4)	227 (14.5)	159 (36.6)	530 (37.6)
Missing	121 (19.2)	304 (15.9)	239 (47.2)	939 (37.5)	486 (52.8)	3193 (69.4)
Triglycerides						
No	188 (40.2)	575 (36.8)	114 (41.9)	696 (38.0)	130 (28.2)	473 (31.0)
Yes	280 (59.8)	989 (63.2)	158 (58.1)	1136 (62.0)	331 (71.8)	1051 (69.0)
Missing	162 (25.7)	350 (18.3)	234 (46.2)	671 (26.8)	460 (49.9)	3077 (66.9)
Hba1c[a]						
No	104 (72.7)	448 (68.1)	52 (63.4)	247 (65.2)	44 (41.1)	127 (39.7)
Yes	39 (27.3)	210 (31.9)	30 (36.6)	132 (34.8)	63 (58.9)	193 (60.3)
**Treatment**						
Antiplatelets						
No	30 (4.8)	82 (4.3)	84 (16.6)	297 (11.9)	68 (7.6)	203 (4.7)
Yes	600 (95.2)	1832 (95.7)	422 (83.4)	2206 (88.1)	825 (92.4)	4097 (95.3)
Missing	0 (0)	0 (0)	0 (0)	0 (0)	28 (3.0)	301 (6.5)
Antihypertensives						
No	27 (4.3)	94 (4.9)	33 (6.5)	243 (9.7)	36 (4.8)	110 (3.3)
Yes	603 (95.7)	1820 (95.1)	473 (93.5)	2260 (90.3)	708 (95.2)	3191 (96.7)
Missing	0 (0)	0 (0)	0 (0)	0 (0)	177 (19.2)	1300 (28.3)
Lipid-lowering medications						
No	53 (8.4)	87 (4.5)	92 (18.2)	615 (24.6)	71 (8.1)	231 (5.4)
Yes	577 (91.6)	1827 (95.5)	414 (81.8)	1888 (75.4)	807 (91.9)	4063 (94.6)
Missing	0 (0)	0 (0)	0 (0)	0 (0)	43 (4.7)	307 (6.7)
Oral hypoglicemics[a]						
No	128 (53.6)	357 (41.7)	87 (38.5)	314 (37.2)	72 (35.1)	122 (25.8)
Yes	111 (46.4)	499 (58.3)	39 (61.5)	530 (62.8)	133 (64.9)	351 (74.2)
Insulin[a]						
No	192 (80.3)	741 (86.6)	151 (66.8)	680 (80.6)	152 (74.1)	365 (77.2)
Yes	47 (19.7)	115 (13.4)	75 (33.2)	164 (19.4)	53 (25.9)	108 (22.8)
Cardiac rehabilitation						
No	439 (69.8)	1423 (74.5)	428 (92.6)	1762 (73.4)	335 (36.7)	918 (20.3)
Yes	190 (30.2)	486 (25.5)	34 (7.4)	638 (26.6)	578 (63.3)	3614 (79.7)
Missing	1 (0.2)	5 (0.3)	44 (8.7)	103 (4.1)	8 (0.9)	69 (1.5)

Footnote: Results are indicated in number of participants (percentages) unless indicated. [a]Values are calculated among diabetic participants. Number of diabetic patients HIC primary (N=921), HIC secondary or tertiary (N=4601), UMIC primary (N=506), UMIC secondary or tertiary (N=2503), LMIC primary (N=630), LMIC secondary or tertiary (N=1914). [b]Risk factor targets are defined as: no smoking or smoking cessation, Moderately vigorous physical activity at least 30 minutes 3-5 times/week, BMI <25 kg/m^2, waist circumference <94 cm in men (<90cm in South-East Asian men) and <80 cm in women, blood pressure <140/90 mmHg (<140/85 mmHg in diabetics), LDL <1.8mmol/L, LDL <1.4mmol/L, non-HDL cholesterol <2.2 mmol/L, triglycerides <1.7 mmol/L, and Hba1c (in diabetic patients) <7%. HICs: high-income counties, UMICs: upper-middle-income countries,

LMICs: lower-middle income countries, CABG: Coronary Artery Bypass Graft, PCI: Percutaneous Coronary Intervention, CVD: cardiovascular disease. [C] Median Hba1c 6.60%.

Supplementary Table 3. SURF CHD II national coordinators, center coordinators, and participating centers by country.

Argentina	Marina Joseph, Yanina Castillo Costa	Socieded Argentina de Cardiología, Buenos Aires
	Nicolás Esteybar Enrique	Counsel of Cardioecology and Healthy Habits Sociedad Argentina de Cardiología, Mar del Plata
Azerbaijan	Rahima Gabulova	Azerbaijan Medical University, Educational Therapeutic Clinic, Baku
	Mahluga Isaveva	Scientific Research Institute for Cardiology, Baku
	Farid Alivev	Baku Health Centre, Baku
	Uzeyir Rahimov	Baku Medical Plaza, Baku
	Galib Imanov	Azerbaijan Medical University, Educational Surgical Clinic, Baku
	Firdovsi Ibrahimov	Central Clinic Hospital, Baku
	Zarbaliyeva Naila	Ganja City United Hospital, Ganja
	Rashad Abasov	ER Medical, Khachmaz
Belgium	Paul Dendale/Anre Jassen	Jessa Hospital-Hartcentrum, Hasselt
	Johan De Sutter	AZ Maria Middelares Cardiologie, Gent
	Sofie Cuypers	Hartcentrum OLV ziekenhuis, Aalst
Brazil	Dalton Precoma	Sociedade Hospitalar Angelina Caron, Campina Grande do Sul, Brazil
	Luiz Ritt	Escola Bahiana de Medicina e Saúde Pública, Salvador, Brazil
	Mario Claudio Soares Sturzeneker	Wallace Thadeu de Mello e Silva Regional University Hospital, Brazil
	Conrado Roberto Hoffmann Filho	Street Blumenau, 294 Centro, Joinville SC
Chile	Maria Teresa Lira	Hospital Clinico Fuerza Aérea de Chile, Santiago
Czech Republic	Michal Varablik, Eva Tumova	Centre of Preventive Cardiology, 3th Department of internal medicine, 1st Faculty of Medicine and General Teaching Hospital, Prague
	Jaromir Ozana	Department of Exercise Medicine and Cardiovascular Rehabilitation, University Hospital Olomouc
Denmark	Ann Bovin	Danish Society of Cardiology, Copenhagen
Estonia	Margus Viigimaa	North Estonia Medical Centre, Tallinn University of Technology, Tallin
Greece	Konstantinos Tsioufis, Ageliki Laina	Cardiology Department, Hippokration General Hospital, Athens
	Zacharoulis Achilles, Fotios Toulgaridis	General Hospital of Athens "Evangelismos", Athens
	Elias Sanidas	LAIKO General Hospital, Athens
Croatia	Zeljko Reiner	Polish Mother's Memorial Hospital Research Institute, Lodz, Poland & University Hospital Center, Zagreb

	Marijana Gulin, Antonijo Bejić	County Hospital Sibenik
	Darko Duplančić	University Hospital, Split
	Jozica Šikić	University Hospital Sveti Duh
Hungary	Eszter Szabados	Ist Department of Medicine, Division of Preventive Cardiology and Rehabilitation, University of Pécs, Medical School, Pécs
Indonesia	Badai Bhatara Tiksnadi	Hasan Sadikin General Hospital, Bandung, West Java
Ireland	Bill McEvoy	University Hospital Galway, Galway
	Anne Reynolds, David Moore	Tallaght University, Hospital Cardiology clinic, Dublin
	Declan Spelman	South Tipperary General Hospital, Tipperary
India	Raman Puri, Rashmi Nanda	Cardiac Care Centre, New Delhi
	Nagaraj Desai	Namana Medical Centre, Bengaluru
	Prabhakar Dorairaj	Ashwin Clinic, Annanagar
	S. S. Iyengar, Sumitra Lakshmana	Manipal Hospital, Bangalore
	Ajay Kumar Pandey	Galaxy Hospital, Mahmoorganj
	Akshyaya Pradhan	King George's Medical University, Lucknow
	Kunal Mahajan	Indira Gandhi Medical College, Shimla
Italy	Pompilio Faggiano	Ospedali Riuniti, Brescia
	P. Zarcone	Policlinico P. Giccone, Palermo
	Maurizio G Abrignani	S Antonio Abate Hospital of Trapani, O.U. of Cardiology, Trapani
Kazakhstan	Makhanov Daniyar	Central Clinical Hospital, Almaty, Kazakhstan
	Kairat Davletov	Asfendiyarov Kazakh National Medical University, Almaty
	Kuatbek Toleubekov, Olga Visternichan	Cardiorehabilitation Center "Tulpar", Karaganda Medical University, Karaganda
	Alibek Mereke	City Policlinic № 32, Al Farabi Kazakh National University, Almaty
	Anar Dushpanova	City Policlinic № 32, Al Farabi Kazakh National University, Almaty
	Bekbolat Zholdin, Zhanat Timirbayev	Medical Center of West Kazakhstan Marat Ospanov Medical University, Zhanakonys
	Gulmira Derbissalina,	Astana Medical University, University hospital , Astana, Kazakhstan
	Daulet K. Aldyngurov	Department of Science and Human resource, Ministry of Healthcare
	Ayan Myssayev,	Cardiological Center of Pavlodar region, Department of Science and Human resourse, Ministry of Healthcare
	Alma Nurtazina	Cardiological Center of Pavlodar region, Semey Medical University, Semey
	Zhanna Romanova, Sundetgali Kalmakhanov	City polyclinic № 5, Al Farabi Kazakh National University, Almaty
Latvia	Iveta Mintale	Latvian Center of Cardiology, Riga
Libya	Omar Msalam	Libyan Cardiac Society, Tripoli

	Emhemed Ehemmali	Misrata Heart & Cardiovascular Center, Misrata
Morocco	Alami Mohamed	Cabinet Cardiologie ALAMI, Casablanca
	Najat Mouine	Cardiology Centre, Mohammed V Military Hospital, Rabat
	Tazi Mezalek Amale	Hopital Universitaire Cheikh Zaid, Rabat
	Aida Soufiani, Zineb Agoumy, Houda Bachri, Imad Massri	Ligue Nationale de cardiologie, Rabat
North Macedonia	Irena Mitevska	University Cardiology Clinic, Skopje
The Netherlands	Saskia Haitjema, Mark de Groot	Utrecht Patient Oriented Database (UPOD), Central Diagnostic Laboratory, Division of Laboratory, Pharmacy, and Biogenetics, University Medical Center Utrecht, Utrecht University, Utrecht
Portugal	Ana Abreu	Hospital Universitário Santa Maria, IMPSP, ISAMB, Faculty of Medicine of University of Lisbon, Lisbon
Romania	Svetlana Mosterou, Dan Gaita	Institute for Cardiovascular Diseases, Timisoara
Russian Federation	Nana Pogosova	National Medical Research Center of Cardiology, Preventive Cardiology Laboratory, Moscow
	Marat V Ezhov	Federal State Budget Institution, National Cardiology Research Centre of Ministry of Healthcare of Russian Federation, Moscow
Saudi Arabia	Abdulhalim Kinsara	Ministry of National Guard health affairs. King Saud bin Abdulaziz University for Health Sciences, COM-WR. King Abdullah International Research Center, Jeddah
Serbia	Ivana Burazor	Cardiology, Institute for rehabilitation; Cardiology, Institute for rehabilitation, and University of Belgrade - Faculty of Medicine, Institute for Cardiovascular Diseases 'Dedinje', Belgrade
	Vojislav Giga	Cardiology Clinic, Clinical Center of Serbia, Belgrade
Spain	Hector Bueno	Hospital 12 de Octubre, Madrid, and Centro Nacional de Investigaciones Cardiovasculares, Madrid
	Regina Dalmau	University Hospital la Paz, Madrid
	Ana García-Barrios	Hospital General Universitario Dr. Balmis, Alicante,
	Jose Antonio Alarcon Duque, Joana Reparaz Mendinueta	Hospital Universitario Donostia/OSI Donostialdea, Donostia
Turkey	Meral Kayikcioglu	Ege University Medical School Cardiology Department, Ege University Tip Fak Kardivoloii AD Bornova Izmir
	Lale Tokgozoglu	Hacettepe University, Department of Cardiology, Ankara
	Emre Aslanger	Yeditepe Univerisity Hospital, Istanbul
	Ayca Turer Cabbar	Yeditepe Univerisity Hospital, Istanbul
United States	Samuel Kim, Caleb Self, Dolores Reynolds,	Weil Cornell Medicine, New York

	Sabrina Rose, Pretti Singh
