# Supplementary material for: A Global Perspective on Cardiovascular Risk Factors by Educational Level in CHD Patients: SURF CHD II
Source: Glob Heart. 2024 Jul 16;19(1):60. doi: 10.5334/gh.1340 (PMC11259115; doi:10.5334/gh.1340)
Supplement: Supplementary File 1. — Sensitivity Analysis. [file gh-19-1-1340-s2.pdf]

SUPPLEMENTARY FILE 2. SENSITIVITY ANALYSIS

Sensitivity analysis by data source

Supplementary Table 4. Participant's characteristics, risk factor recording, target attainment, and treatment by data source.

	Pre-existing registry (N=4730)	Patient interview and medical records (N=9154)
Mean age (SD)	66.7 (11.1)	63.6 (11.1)
Seks		
Female	1217 (25.7)	2248 (24.6)
Male	3513 (74.3)	6906 (75.4)
Educational level		
Primary school	473 (11.9)	1584 (17.3)
Secondary school	439 (11.0)	3379 (36.9)
Tertiary	2656 (66.8)	2544 (27.8)
Unknown	411 (10.3)	1642 (17.9)
**Recording**		
Smoking		
No	351 (7.4)	255 (2.8)
Yes	4379 (92.6)	8899 (97.2)
Phsyical activity		
No	3225 (68.2)	1125 (12.3)
Yes	1505 (31.8)	8029 (87.7)
Systolic BP		
No	458 (9.7)	516 (5.6)
Yes	4272 (90.3)	8638 (94.4)
Diastolic BP		
No	458 (9.7)	535 (5.8)
Yes	4272 (90.3)	8619 (94.2)
BMI		
No	3281 (69.4)	2402 (26.2)
Yes	1449 (30.6)	6752 (73.8)
Waist circumference		
No	4300 (90.9)	6507 (71.1)
Yes	430 (9.1)	2647 (28.9)
Total cholesterol		
No	3967 (83.9)	1870 (20.4)
Yes	763 (16.1)	7284 (79.6)
LDL Cholesterol		
No	780 (16.5)	2204 (24.1)
Yes	3950 (83.5)	6950 (75.9)
HDL cholesterol		
No	4013 (84.8)	2512 (27.4)
Yes	717 (15.2)	6642 (72.6)
Triglycerides		
No	3967 (83.9)	2322 (25.4)
Yes	763 (16.1)	6832 (74.6)
Hba1c[a]		
No	192 (67.6)	1403 (42.8)

Yes	92 (32.4)	1872 (57.2)
**Target attainment[b]**		
Never or former smoking		
No	1042 (23.8)	1783 (20.0)
Yes	3337 (76.2)	7116 (80.0)
Physical activity		
No	696 (46.2)	3613 (45.0)
Yes	809 (53.8)	4416 (55.0)
BMI		
No	1323 (70.9)	4947 (73.5)
Yes	543 (29.1)	1781 (26.5)
Waist circumference		
No	360 (83.7)	2227 (84.2)
Yes	70 (16.3)	418 (15.8)
BP		
No	1401 (76.2)	3385 (39.2)
Yes	438 (23.8)	5243 (60.8)
LDL <1.8mmol/L		
No	2006 (50.8)	4451 (64.0)
Yes	1944 (49.2)	2499 (36.0)
LDL <1.4mmol/L		
No	2994 (75.8)	5638 (81.1)
Yes	956 (24.2)	1312 (18.9)
Non-HDL cholesterol		
No	386 (60.9)	4525 (69.2)
Yes	248 (39.1)	2011 (30.8)
Triglycerides		
No	223 (29.2)	2439 (35.7)
Yes	540 (70.8)	4393 (64.3)
Hba1c[a]		
No	34 (37.0)	1135 (60.6)
Yes	58 (63.0)	737 (39.4)
**Treatment**		
Antiplatelet		
No	177 (4.1)	817 (8.9)
Yes	4145 (95.9)	8337 (91.1)
Antihypertensives		
No	42 (1.4)	671 (7.3)
Yes	3031 (98.6)	8483 (92.7)
Lipid-lowering		
No	271 (6.3)	1171 (12.8)
Yes	4036 (93.7)	7983 (87.2)
Oral hypoglicemics[a]		
No	127 (44.7)	1254 (38.3)
Yes	157 (55.3)	2021 (61.7)
Insulin[a]		
No	1448 (90.8)	8447 (92.3)
Yes	147 (9.2)	707 (7.7)
Cardiac rehabilitation		
No	167 (58.8)	2603 (79.5)

Yes	117 (41.2)	672 (20.5)

Footnote: Results are indicated in percentages unless indicated. [a] Percentages are provided among diabetic patients. Number of diabetic patients was 284 (pre-existing registry) and 3275 (interview). [b] Risk factor targets are defined as: no smoking or smoking cessation, Moderately vigorous physical activity at least 30 minutes 3-5 times/week, BMI <25 kg/m^2, waist circumference <94 cm in men (<90cm in South-East Asian men) and <80 cm in women, blood pressure <140/90 mmHg (<140/85 mmHg in diabetics), LDL <1.8mmol/L, LDL <1.4mmol/L, non-HDL cholesterol <2.2 mmol/L, triglycerides <1.7 mmol/L, and Hba1c (in diabetic patients) <7%.

Supplementary Table 5. Results multivariable analysis showing odds ratios for achieving risk factor targets, being on medication or participate in cardiac rehabilitation, if having secondary or tertiary educational level compared to primary education, stratified by data source.

	**Pre-existing registry**	**Patient interview and medical records**
**Target attainment[a]**		
Never or former smoking	2.01 (1.58-2.55)	1.60 (1.38-1.85)
Physical activity	1.65 (1.12-2.47)	1.47 (1.30-1.66)
BMI	0.94(0.60-1.52)	0.80 (0.70-0.93)
Blood pressure		0.91 (0.80-1.02)
LDL <1.8mmol/L	1.08 (0.86-1.36)	1.02 (0.89-1.18)
LDL <1.4mmol/L	0.87 (0.67-1.13)	0.91 (0.77-1.08)
Non-HDL cholesterol		0.97 (0.84-1.13)
Tryglicerides	0.42 (0.08-1.63)	1.09 (0.95-1.25)
**Treatment**		
Antiplatelets		1.16 (0.96-1.40)
Antihypertensives		0.88 (0.69-1.11)
Lipid-lowering		0.74 (0.62-0.88)
Cardiac rehabilitation		1.38 (1.21-1.58)

Footnote: Results are expressed in odds ratios (95% confidence intervals) of achieving risk factor targets, and being on medication or participate in cardiac rehabilitation, if having secondary or tertiary education compared to primary education, adjusted by age,

sex and type of center (public or private). [a] Risk factor targets are defined as: no smoking or smoking cessation, Moderately vigorous physical activity at least 30 minutes 3-5 times/week, BMI <25 kg/m2, waist circumference <94 cm in men (<90cm in South-East Asian men) and <80 cm in women, blood pressure <140/90 mmHg (<140/85 mmHg in diabetics), LDL <1.8mmol/L, LDL <1.4mmol/L, non-HDL cholesterol <2.2 mmol/L, triglycerides <1.7 mmol/L. Empty cells indicate that models could not be fitted due to lack of observations.

Sensitivity analysis by CHD diagnostic category

Supplementary Table 6. Participant's characteristics, risk factor recording, target attainment, and treatment by data source.

	CABG(N=4289)	PCI (N=8704)	SAP (N=4540)	ACS (N=6624)
**Patient characteristics**				
Mean age (SD)	67.2 (9.91)	64.1 (11.2)	65.4 (10.9)	63.9 (11.5)
Sex				
Female	401 (21.0%)	1429 (22.6)	1266 (27.9)	1575 (23.8)
Male	1509 (79.0)	4896 (77.4)	3274 (72.1)	5049 (76.2)
Educational level				
Primary school	298 (16.0)	952 (15.8)	625 (15.2)	1064 (16.5)
Secondary school	649 (34.9)	2115 (35.1)	1138 (27.6)	1731 (26.8)
Tertiary	599 (32.2)	1899 (31.5)	1674 (40.6)	2687 (41.6)
Unknown	312 (16.8)	1056 (17.5)	687 (16.7)	980 (15.2)
**Recording**				
Smoking				
No	72 (3.8)	360 (5.7)	226 (5.0)	259 (3.9)
Yes	1838 (96.2)	5965 (94.3)	4314 (95.0)	6365 (96.1)
Phsyical activity				
No	271 (14.2)	1161 (18.4)	1716 (37.8)	2401 (36.2)
Yes	1639 (85.8)	5164 (81.6)	2824 (62.2)	4223 (63.8)
Systolic BP				
No	77 (4.0)	390 (6.2)	639 (14.1)	282 (4.3)
Yes	1833 (96.0)	5935 (93.8)	3901 (85.9)	6342 (95.7)
Diastolic BP				
No	80 (4.2)	401 (6.3)	646 (14.2)	283 (4.3)
Yes	1830 (95.8)	5924 (93.7)	3894 (85.8)	6341 (95.7)

BMI				
No	485 (25.4)	1853 (29.3)	2098 (46.2)	2911 (43.9)
Yes	1425 (74.6)	4472 (70.7)	2442 (53.8)	3713 (56.1)
Waist circumference				
No	1314 (68.8)	4498 (71.1)	3838 (84.5)	5178 (78.2)
Yes	596 (31.2)	1827 (28.9)	702 (15.5)	1446 (21.8)
Total cholesterol				
No	483 (25.3)	1486 (23.5)	2114 (46.6)	3092 (46.7)
Yes	1427 (74.7)	4839 (76.5)	2426 (53.4)	3532 (53.3)
LDL Cholesterol				
No	445 (23.3)	1413 (22.3)	1184 (26.1)	1111 (16.8)
Yes	1465 (76.7)	4912 (77.7)	3356 (73.9)	5513 (83.2)
HDL cholesterol				
No	585 (30.6)	1869 (29.5)	2353 (51.8)	3317 (50.1)
Yes	1325 (69.4)	4456 (70.5)	2187 (48.2)	3307 (49.9)
Triglycerides				
No	523 (27.4)	1598 (25.3)	2357 (51.9)	3200 (48.3)
Yes	1387 (72.6)	4727 (74.7)	2183 (48.1)	3424 (51.7)
Hba1c (among diabetics)				
No	303 (38.1)	928 (43.9)	614 (43.0)	539 (49.4)
Yes	493 (61.9)	1184 (56.1)	813 (57.0)	552 (50.6)
**Target attainment**				
Never or former smoking				
No	247 (13.4)	1231 (20.6)	772 (17.9)	1696 (26.6)
Yes	1591 (86.6)	4734 (79.4)	3542 (82.1)	4669 (73.4)
Physical activity				
No	848 (51.7)	2341 (45.3)	1272 (45.0)	1891 (44.8)
Yes	791 (48.3)	2823 (54.7)	1552 (55.0)	2332 (55.2)
BMI				
No	1064 (74.8)	3308 (73.3)	2044 (73.3)	2708 (72.8)
Yes	359 (25.2)	1207 (26.7)	746 (26.7)	1011 (27.2)
Waist circumference				
No	509 (85.4)	1589 (87.0)	613 (87.3)	1212 (83.9)
Yes	87 (14.6)	237 (13.0)	89 (12.7)	233 (16.1)
BP				
No	759 (42.7)	2163 (37.9)	1562 (49.8)	2098 (44.6)
Yes	1020 (57.3)	3547 (62.1)	1572 (50.2)	2602 (55.4)
LDL <1.8mmol/L				
No	851 (58.1)	2925 (59.5)	2194 (65.4)	3252 (59.0)
Yes	614 (41.9)	1987 (40.5)	1162 (34.6)	2261 (41.0)
LDL <1.4mmol/L				
No	1141 (77.9)	3875 (78.9)	2817 (83.9)	4356 (79.0)

Yes	324 (22.1)	1037 (21.1)	539 (16.1)	1157 (21.0)
Non-HDL cholesterol				
No	877 (67.3)	2916 (66.5)	1599 (75.1)	2230 (69.4)
Yes	426 (32.7)	1472 (33.5)	531 (24.9)	984 (30.6)
Triglycerides				
No	474 (34.2)	1555 (32.9)	840 (38.5)	1182 (34.5)
Yes	913 (65.8)	3172 (67.1)	1343 (61.5)	2242 (65.5)
Hba1c (among diabetics)				
No	312 (63.3)	683 (57.7)	475 (58.4)	334 (60.5)
Yes	181 (36.7)	501 (42.3)	338 (41.6)	218 (39.5)
**Treatment**				
Antiplatelet				
No	196 (10.3)	403 (6.4)	368 (8.3)	380 (6.0)
Yes	1713 (89.7)	5918 (93.6)	4040 (91.7)	5971 (94.0)
Antihypertensives				
No	99 (5.2)	389 (6.3)	225 (5.7)	218 (3.9)
Yes	1793 (94.8)	5832 (93.7)	3739 (94.3)	5339 (96.1)
Lipid-lowering				
No	193 (10.1)	560 (8.9)	649 (14.7)	479 (7.6)
Yes	1715 (89.9)	5758 (91.1)	3761 (85.3)	5855 (92.4)
Oral hypoglicemics				
No	275 (34.5)	796 (37.7)	550 (38.5)	462 (42.3)
Yes	521 (65.5)	1316 (62.3)	877 (61.5)	629 (57.7)
Insulin				
No	598 (75.1)	1638 (77.6)	1071 (75.1)	861 (78.9)
Yes	198 (24.9)	474 (22.4)	356 (24.9)	230 (21.1)
Cardiac rehabilitation				
No	933 (51.3)	3792 (65.0)	1908 (48.8)	2718 (42.8)
Yes	884 (48.7)	2046 (35.0)	2002 (51.2)	3628 (57.2)

Footnote: Results are indicated in percentages unless indicated. [a] Percentages are provided among diabetic patients. Number of diabetic patients was 796 (CABG), 2112 (PCI), 1427 (ACS), and 1091 (SAP). [b] Risk factor targets are defined as: no smoking or smoking cessation, Moderately vigorous physical activity at least 30 minutes 3-5 times/week, BMI <25 kg/m^2, waist circumference <94 cm in men (<90cm in South-East Asian men) and <80 cm in women, blood pressure <140/90 mmHg (<140/85 mmHg in

diabetics), LDL <1.8mmol/L, LDL <1.4mmol/L, non-HDL cholesterol <2.2 mmol/L, triglycerides <1.7 mmol/L, and Hba1c (in diabetic patients) <7.

Supplementary Table 7. Results multivariable analysis showing odds ratios for achieving risk factor targets, being on medication or participate in cardiac rehabilitation, if having secondary or tertiary educational level compared to primary education, stratified by CHD diagnostic category.

	CABG	PCI	ACS	SAP
**Target attainment**				
Never or former smoking	2.18 (1.45-3.23)	1.45 (1.20-1.75)	1.57 (1.33-1.85)	1.74 (1.38-2.19)
Physical activity	2.10 (1.56-2.84)	1.76 (1.50-2.08)	1.69 (1.43-2.00)	1.19 (0.96-1.48)
BMI	0.93 (0.66-1.33)	0.82 (0.68-1.00)	0.84 (0.69-1.03)	0.73 (0.57-0.94)
Blood pressure	0.74 (0.39-1.48)	0.63 (0.44-0.94)	0.61 (0.42-0.91)	0.60 (0.33-1.14)
LDL <1.8mmol/L	1.17 (0.83-1.65)	1.25 (1.05-1.50)	1.28 (1.09-1.50)	1.118 (0.90-1.39)
LDL <1.4mmol/L	1.18 (0.78-1.80)	0.99 (0.80-1.23)	1.02 (0.84-1.24)	1.07 (0.81-1.44)
Non-HDL cholesterol	1.39 (0.95-2.06)	1.06 (0.88-1.29)	0.96 (0.77-1.19)	0.98 (0.74-1.30)
Tryglicerides	1.83 (1.30-2.58)	1.05 (0.88-1.27)	1.06 (0.87-1.30)	0.99 (0.76-1.27)
Hba1c [b]	1.40 (0.81-2.48)	1.24 (0.90-1.70)	2.02 (1.56-2.61)	1.28 (0.90-1.82)
**Treatment**				
Antiplatelets	1.36 (0.89-2.02)	1.10 (0.83-1.46)	1.16 (0.87-1.53)	1.36 (1.00-1.85)
Antihypertensives	0.89 (0.45-1.64)	0.76 (0.54-1.06)	0.94 (0.62-1.39)	1.06 (0.68-1.58)
Lipid-lowering	0.81 (0.50-1.26)	0.83 (0.64-1.07)	1.06 (0.81-1.36)	0.77 (0.58-1.00)
Oral hypoglicemics [b]	1.58 (1.03-2.41)	1.22 (0.95-1.56)	1.30 (0.98-1.74)	1.29 (0.92-1.81)
Insulin [b]	0.51 (0.32-0.83)	0.64 (0.48-0.86)	0.72 (0.52-1.00)	0.78 (0.51-1.20)
Cardiac rehabilitation	1.58 (1.20-2.10)	1.85 (1.55-2.22)	2.84 (2.44-3.30)	1.46 (1.20-1.77)

Footnote: Results are expressed in odds ratios (95 confidence intervals) of achieving risk factor targets, and being on medication or participate in cardiac rehabilitation, if having secondary or tertiary education compared to primary education, adjusted by age , sex and type of center (public or private) [a] Risk factor targets are defined as: no smoking or smoking cessation, Moderately vigorous physical activity at least 30 minutes 3-5 times/week, BMI <25 kg/m2, waist circumference <94 cm in men (<90cm

in South-East Asian men) and <80 cm in women, blood pressure <140/90 mmHg (<140/85 mmHg in diabetics), LDL <1.8mmol/L, LDL <1.4mmol/L, non-HDL cholesterol <2.2 mmol/L, triglycerides <1.7 mmol/L, and Hba1c (in diabetic patients) <7. [b] Estimates are calculated among diabetic patients.
